# Integrated multi-omics of the gastrointestinal microbiome and ruminant host reveals metabolic adaptation underlying early life development

**DOI:** 10.1186/s40168-022-01396-8

**Published:** 2022-12-12

**Authors:** Xiaoting Yan, Huazhe Si, Yuhang Zhu, Songze Li, Yu Han, Hanlu Liu, Rui Du, Phillip B. Pope, Qiang Qiu, Zhipeng Li

**Affiliations:** 1 College of Animal Science and Technology, Jilin Agricultural University, Changchun, 130118 China; 2 School of Ecology and Environment, Northwestern Polytechnical University, Xi’an, 710100 China; 3 Department of Special Animal Nutrition and Feed Science, Institute of Special Animal and Plant Sciences, Chinese Academy of Agricultural Sciences, Changchun, 130112 China; 4 Jilin Provincial Engineering Research Center for Efficient Breeding and Product Development of Sika Deer, Changchun, 130118 China; 5 Key Lab of Animal Production, Product Quality and Security, Ministry of Education, Jilin Agricultural University, Changchun, 130118 China; 6 College of Chinese Medicine Materials, Jilin Agricultural University, Changchun, 130118 China; 7 Faculty of Biosciences, Norwegian University of Life Sciences, 1433 Ås, Norway; 8 Faculty of Chemistry, Biotechnology and Food Science, Norwegian University of Life Sciences, 1433 Ås, Norway

**Keywords:** Early life, Fatty acid metabolism, Cooperation, Region- and stage-specific development, Ontogeny, Butyrate, Immune response

## Abstract

**Background:**

The gastrointestinal tract (GIT) microbiome of ruminants and its metabolic repercussions vastly influence host metabolism and growth. However, a complete understanding of the bidirectional interactions that occur across the host-microbiome axis remains elusive, particularly during the critical development stages at early life. Here, we present an integrative multi-omics approach that simultaneously resolved the taxonomic and functional attributes of microbiota from five GIT regions as well as the metabolic features of the liver, muscle, urine, and serum in sika deer (*Cervus nippon*) across three key early life stages.

**Results:**

Within the host, analysis of metabolites over time in serum, urine, and muscle (*longissimus lumborum*) showed that changes in the fatty acid profile were concurrent with gains in body weight. Additional host transcriptomic and metabolomic analysis revealed that fatty acid *β*-oxidation and metabolism of tryptophan and branched chain amino acids play important roles in regulating hepatic metabolism. Across the varying regions of the GIT, we demonstrated that a complex and variable community of bacteria, viruses, and archaea colonized the GIT soon after birth, whereas microbial succession was driven by the cooperative networks of hub populations. Furthermore, GIT volatile fatty acid concentrations were marked by increased microbial metabolic pathway abundances linked to mannose (rumen) and amino acids (colon) metabolism. Significant functional shifts were also revealed across varying GIT tissues, which were dominated by host fatty acid metabolism associated with reactive oxygen species in the rumen epithelium, and the intensive immune response in both small and large intestine. Finally, we reveal a possible contributing role of necroptosis and apoptosis in enhancing ileum and colon epithelium development, respectively.

**Conclusions:**

Our findings provide a comprehensive view for the involved mechanisms in the context of GIT microbiome and ruminant metabolic growth at early life.

Video Abstract

**Supplementary Information:**

The online version contains supplementary material available at 10.1186/s40168-022-01396-8.

## Introduction

Cumulative evidence continues to underscore that the diverse and complex microbiome residing in gastrointestinal tract (GIT) is a fundamental factor influencing ruminant productivity and associated milk and meat commodities [1]. Importantly, it is well recognized that microbial colonization of the GIT after birth [2] has long-lasting impacts on the development of animal microbiome [3] and the health and phenotype of the host into adulthood [3–5]. For example, the microbiome and its metabolites are known to regulate papillae morphology and growth of the rumen epithelium of both cattle and sheep [6, 7]. Butyrate-producing bacteria have also been shown to be associated with the regulation of host immunity and metabolism in the hindgut of cattle [4]. Indeed, manipulating GIT microbiome at early life also produced a persistent and long-termed effect on young ruminants [8].

For young ruminants, the most dramatic physiological challenges are the events surrounding the development of the rumen that could be classified into three key stages: non-rumination phase (0–21 days), transition phase (21–56 days), rumination phase (from 56 days onwards) [9]. Rumination entails the successful smooth transition from a milk-based liquid to solid forages, which concomitantly results in a major shift in the pattern of nutrients being delivered to the varying GIT regions [10], the types and the functions of the that live there [11], host’s gene expression [12], and serum metabolic profiles [13]. This collective change across the “host-microbiome axis” during the birth to postweaning transition leads to principle of metabolic transformations that occur beyond the GIT, particularly in the liver, which shifts from glycolytic metabolism (glucose supplied from milk) to glucogenic metabolism (volatile fatty acid supplied from the microbiota) that is ultimately reflected in the growth and development of the host [10]. Given the connectivity between microbiome and host metabolism throughout early life, there is a need for joint analysis that takes into account the relationship between the GIT microbiome and metabolites as well as host features important in ruminant growth such as epithelial and hepatic functions, and serum, urine, and body composition.

While cattle, sheep, and goats are commonly used as models for examining juvenile ruminant development, sika deer (*Cervus nippon*) are a valuable economic and medical resource, being farmed for velvet antler and their high-quality meat and skin. Differing to the grass and roughage eaters, such as cattle and sheep, sika deer are classified as intermediate opportunistic feeders, which have the enlarged absorptive surface of rumen papilla [14]. These traits make the sika deer an excellent organism for investigating development of GIT microbiome and its relationship to variation in host growth. To better understand interactions across the host-microbiome axis at a deeper molecular level and how it develops during critical stages of early life, this study aimed to co-analyze and visualize both host metabolism as well as microbiome structure and function simultaneously. To meet our objectives, a combination of GIT metagenomics, transcriptomics (GIT epithelium and liver), and both untargeted (GIT lumen, serum, urine, liver) and targeted metabolomics (*longissimus lumborum*, *LL*) was employed on juvenile sika deer after birth at day 1 (after birth), day 42 (preweaning), and day 70 (postweaning). Our subsequent integrative multi-omics analysis aimed to reveal insight into the following: (i) the variation of body composition and the overall metabolic phenotypes in juvenile growth, (ii) hepatic metabolism response during early life ruminant development, and (ii) how the GIT microbial community and functional capacity assembles and maintains, in concert with epithelial function, across the longitudinal GIT axis.

## Results and discussion

### Significant change of fatty acid profiles in body composition, serum, and urine

**Fig. 1:**
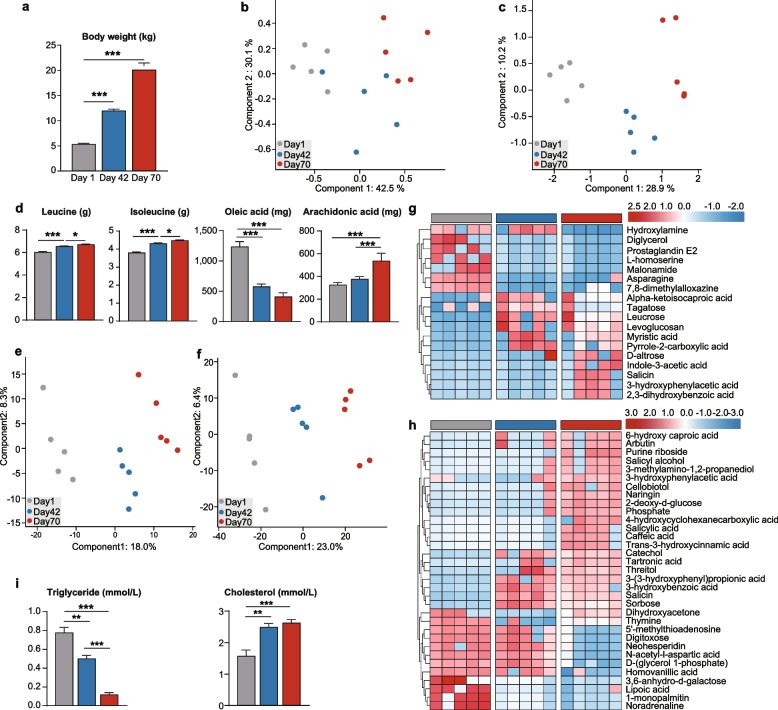
A metabolic view and signature of sika deer from birth to postweaning. **a** The column chart revealing the growth of whole-body wet weight of sika deer at days 1 (gray), 42 (blue), and 70 (red). PLS-DA showing the shift of amino acids (**b**) and fatty acids (**c**) in *longissimus lumborum* (*LL*) of sika deer among the three stages. **d** Box plots showing 4 metabolites in *LL* significantly changed during early growth period. Benjamini-Hochberg-adjusted *P*-values were determined by ANOVA. Bar and whiskers represent the mean ± s.d. PLS-DA and heat map revealing the changed metabolites in serum (**e** and **g**) and urine (**f** and **h**). Metabolite samples at days 1, 42, and 70 in the PLS-DA plot are colored by gray, blue, and red circles, respectively. The metabolites differentially expressed in heat map were identified by VIP values (> 1), SAM, and/or ANOVA methods. **i** The significantly changed concentrations of the triglyceride and cholesterol in serum. The gray, blue, and red bars at top of heat map represent the samples at days 1, 42, and 70, respectively. *, **, and *** indicate the Benjamini-Hochberg-adjusted *P*-value < 0.05, < 0.01, and < 0.001, respectively

To evaluate the body growth trait of juvenile sika deer after birth (day 1), preweaning (day 42), and postweaning (day 70), we firstly found the significant and linear increase of body weight, which achieved a weight gain 4-fold higher at day 72 than that at day 1 (Fig. 1a), indicating an increased nutritional deposition after birth. Because weight gain is closely associated with amino acid (AA) and fatty acid (FA) availability in support of growth, we subsequently determined the concentrations of AA and FA in *longissimus lumborum* (*LL*). We found a striking stage-dependent clustering effect of FA concentrations (Figs. 1c and S1a–b) but minimal clustering for AAs (Figs. 1b and S2 a–b), suggesting that the metabolic profile of fatty acids was significantly regulated during growth. Further comparative analysis showed that the concentrations of aspartate, lysine, leucine, and isoleucine, and arachidonic acid linearly increased, whereas oleic acid and cis-10-heptadecenoic acid decreased in *LL* from birth to postweaning (Figs. 1d, S1c, and S2c). Observed increases in leucine levels agreed with leucine being recognized as a nutrient signal stimulating protein synthesis in skeletal muscle [15]. Oleic acid was the most abundant fatty acid observed in ruminant muscle [16], which is produced by the *Δ*9 desaturation of stearic acid, and its decrease over time suggested lower desaturation activity. In contrast, observed increases in the polyunsaturated acid, arachidonic acid (n-6 PUFA), was also consistent with previous demonstrations that acids of the n-6 PUFA family serve as an important regulating factor for growth in early postnatal life [17]. Collectively, these results indicated an apparent increase in saturation and a corresponding decrease in unsaturation of fatty acids of muscle with age, suggesting a possible relationship between the decreased activity by fatty acid desaturase and muscle tissue with age.

To further understand host metabolism phenotypes that are associated with growth, we characterized the metabolites in serum and urine of sika deer using gas chromatography-mass spectrometry (GC-MS). The partial least squares-discriminant analysis (PLS-DA) showed that the metabolites in serum (Figs. 1e, S3a–b) and urine (Figs. 1f, S4a–b) were clearly distinct among the three stages, with a pronounced separation between preweaning and postweaning stages in urine (Fig. S4 a–b), suggesting that the urinary metabolites reflect the varying physiological processes that occur during early life transition.

Subsequently, we identified a total of 18 and 33 metabolites that were significantly changed in serum (Figs. 1g and S3c) and urine (Figs. 1h and S4c), respectively, based on variable importance in projection (VIP) scores (> 1.0), SAM, and/or ANOVA. Concentrations of caffeic acid and its derivative (trans-3-hydroxycinnamic acid) in urine (Fig. 1h), and leucrose in serum (Fig. 1g) increased with age, both of which have been linked to increases in FA *β*-oxidation, and significantly inhibition of fatty acid accumulation [18, 19]. The concentration of 1-monopalmitin, an intraluminal hydrolysis product of triglyceride in milk [20], significantly decreased in urine with age indicating the pattern of fat digestion and absorption changed with animal growth. Furthermore, the phenolic acids (3-hydroxyphenylacetic acid, 3-hydroxybenzoic acid, 3-(3-hydroxyphenyl) propionic acid, 3-hydroxycinnamic acid) increased in both serum and urine (Fig. 1g and h), which has been documented to affect the cholesterol metabolism [21]. Meanwhile, myristic acid, well-known predictor of serum cholesterol [22], also increased over time in serum, whereas the concentrations of triglyceride and cholesterol significantly decreased and increased in serum, respectively (Fig. 1i). Our results suggested an increased efficiency of FA oxidation and cholesterol production resulting in the change of body composition phenotype.

### Global transcriptome and metabolome revealing change of hepatic fatty acid metabolism

**Fig. 2:**
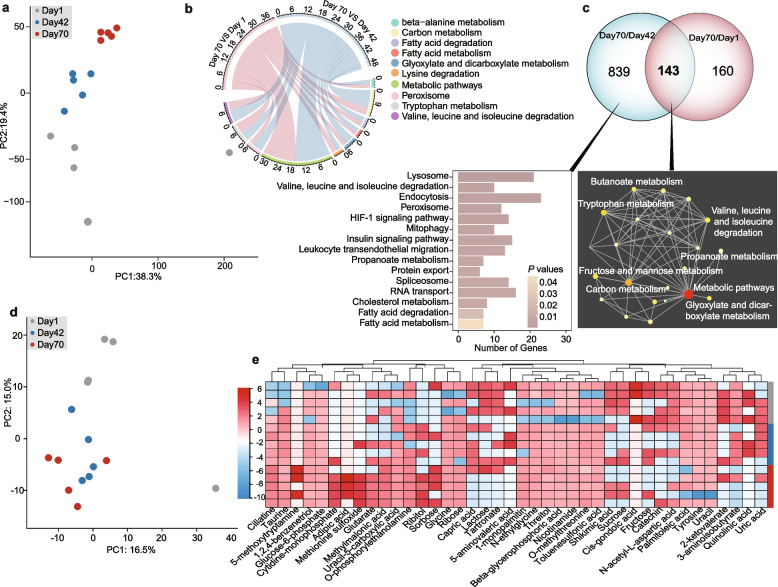
Global transcriptional and metabolic shift in the liver of sika deer from birth to postweaning. **a** PCA of all hepatic expressed genes. PCA vector separates samples into age groups and is colored by gray, blue, and red circles, respectively. **b** A circular plot showing 10 significantly enriched pathways of the upregulated DEGs in liver. The DEGs were determined by the fold change ≥ 2 and a Benjamini-Hochberg-adjusted *P*-value < 0.05. The pink and light blue curves represent the comparison of day 70 vs day 1 and day 70 vs day 42, respectively. The number at out circle indicates the size of the gene sets in each pathway. Pathway enrichment analysis was performed by a one-side Fisher test based on the pathway annotations (full results were supplied in Table S2). **c** Comparison of the overlapped and specific upregulated DEGs and the enriched pathways in the liver. The pink and light blue circles represent the comparison of day 70 vs day 1 and day 70 vs day 42, respectively. **d** PCA of all sika deer hepatic metabolome samples. PCA vector separates samples into age groups and is colored by gray, blue and red circles, respectively. **e** PCA of the global metabolome liver are colored (gray to blue to red) to the age groups. **f** Heat map of metabolites differentially expressed across the different time points in the liver, constrained to the significant metabolites identified by VIP values (> 1), SAM, and/or ANOVA methods. Colors indicate the normalized relative concentration of each metabolite from minimum (blue) to maximum (red). From top to bottom: day 1: gray, day 42: blue, and day 70: red

To understand the metabolic adaptation of the liver that occurs during animal growth, we conducted RNA sequencing-based transcriptome and untargeted metabolome analysis. The principal component analysis (PCA) based on the annotated genes (Table S1) showed that the global liver gene expression at postweaning (day 70) was clearly different from that at birth (day 1) and preweaning (day 42), with 982 differently expressed genes (DEGs) were upregulated at day 70 vs day 42, whereas 303 DEGs were upregulated and 203 were downregulated at day 70 vs day 1 (Fig. 2a, Supplementary Discussion). Across all time points, the 982 and 303 upregulated DEGs were enriched in 44 and 13 metabolic pathways (Table S2). Hepatic metabolomics supported our RNA-seq observations, with a total of 40 specific metabolites significantly variant at day 70 compared to days 1 and 42 (Figs. 2d–e, S5b-c). These results collectively indicated that the liver of juvenile sika deer undergoes a dramatic shift at early life, which is most likely reflected by changes in the dietary substrates metabolized, transforming from glucose and long-chain fatty acids after birth to volatile fatty acids (VFAs), and ketones after rumen transformation [10]. It is note that secondary compounds such as tannins, essential oils, and flavonoids are also widespread in plants, which play a role on ruminant digestion and product quality [23]. Thus, the significantly increased hepatic metabolites would also result from the ingested plant secondary compounds in diet.

We further revealed that the upregulated DEGs were commonly enriched in beta-alanine metabolism, fatty acid metabolism, valine, leucine and isoleucine degradation, and tryptophan metabolism (Fig. 2b and Table S2). Moreover, the shared 143 upregulated DEGs (day 70 vs day 42 and day 70 vs day 1) were also enriched in the tryptophan metabolism, valine, leucine, and isoleucine degradation (Figs. 2c and S5a). Tryptophan has been shown to act as an inhibitor of gluconeogenesis in the liver [24], whereas branched chain amino acids (BCAAs: leucine, isoleucine, and valine) trigger glycine use as a carbon donor for the pyruvate-alanine cycle regulating lipid homeostasis [25]. Supporting in our data, the concentrations of glycine and glucose-6-phosphate increased with animal growth (Fig. 2e). These results suggested that tryptophan and BCAAs are likely to play a critical role in regulating liver metabolic transition during early life of ruminants.

In addition, several genes associated with transport of lipid and FA oxidation were upregulated, including *Ehhadh*, *Echs1*, and *Hmgcs2* (Fig. S5a). *Ehhadh* is part of the classical peroxisomal FA *β*-oxidation pathway, which regulates dicarboxylic acids metabolism and hepatic cholesterol biosynthesis [26], whereas *Echs1* and *Hmgcs2* are key genes in FA *β*-oxidation involved in the metabolism of fatty acyl-CoA esters and ketogenesis [27, 28]. Moreover, the concentrations of adipic acid and glutarate (dicarboxylic acids) were increased with animal growth (Fig. 2e). Our results indicated that FA *β*-oxidation was identified as an important biological function in affecting hepatic lipid homeostasis at early life.

To further reveal the changes from pre to postweaning, we found that the 839 upregulated DGEs (day 70 and day 42) were those enriched in lysosome, endocytosis, mitophagy, and cholesterol metabolism in hepatic functions (Fig. 2c). This specifically included genes, such as *Lipa*, *Lamp2*, *Ldlr*, *Npc2*, *Acaa2*, *Acsl*, and *Cpt1* (Table S2, Supplementary Discussion), regulating cholesterol transport and efflux [29], and release and transportation of long-chain FAs into the mitochondria for FA *β*-oxidation [30–32]. Lysosomes promote lipid catabolism and transport, both of which are critical in maintaining cellular lipid homeostasis [33]. Our results show that cholesterol transport and metabolism for energy production via FA *β*-oxidation in the liver are likely enhanced from preweaning to postweaning for juvenile sika deer, which could represent a significant source for the changes in FA composition that were observed in *LL* (Fig. 1c and i, Supplementary Discussion).

### Immediately diverse and regional microbe colonization across GIT after birth

**Fig. 3:**
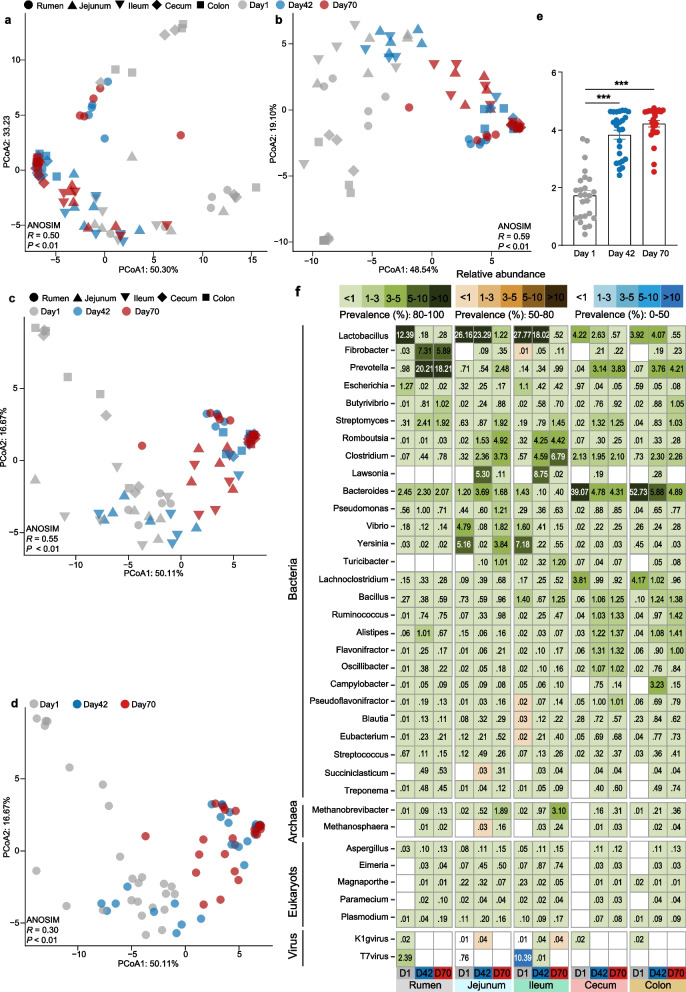
Regional taxonomic differences of GIT microbiota of sika deer from birth to postweaning. PCoA of GIT taxonomic community composition at phylum (**a**), family (**b**), and genus levels (**c**, **d**) based on Bray-Curtis dissimilarity. The microbial samples from GIT regions were indicated as different shapes (rumen: circle, jejunum: triangle, ileum: inverted triangle, cecum: rhombus, and colon: square), and different time points were indicated by filling color (day 1: gray, day 42: blue, day 70: red) in **a**, **b**, and **c**. ANOSIM analysis was used for statistical testing of group similarities. The proportion of variation explained for each axis is given after colon. **e** Bar plot revealing the Shannon diversity index among the three age groups. The diversity index was calculated using the taxonomic composition at genus level. Bar and whiskers represent the mean ± s.d. **f** Relative abundances of bacteria, archaea, eukaryotes, and virus, as averaged over all samples (*n* = 5) in each GIT regions for each time points, are given as percentages (× 100). Prevalence heat map indicates the proportion of any specific taxonomy observed in all samples. D, day. *** indicates the Benjamini-Hochberg-adjusted *P*-value < 0.001

Since a conventional GIT microbiome is necessary for nutrient metabolism and animal growth after birth, we systematically examined rumen, jejunum, ileum, cecum, and colon microbiome structure and function using shotgun metagenomic sequencing. From a total of 196 Gb high-quality reads, a total of 4,054,139 contigs with N50 length of 1617 bp were assembled including 7,131,184 predicted ORFs (Table S3). Analysis of the microbial composition features identified bacteria (93.59%), eukaryotes (2.36%), archaea, and viruses. Taxonomic assignment highlighted that the bacterial phyla Bacteroidetes, Proteobacteria and Firmicutes were dominant (Fig. 3f, Supplementary Discussion), and a low abundance of archaea (*Methanobrevibacter* spp.), and fungi (*Aspergillus* spp. and *Magnaporthe* spp.) were observed in the GIT after birth, consisting with previous findings of calves and goats [2, 5–7, 34].

Principal coordinates analysis (PCoA) showed that a progressive and heterogeneous establishment of GIT microbial populations occurs during early life (Fig. 3). In particular, the GIT microbial composition at a phylum (Figs. 3a and S6 a–b), family (Figs. 3b and S6 c–d), and genus (Figs. 3c–d and S6 e–f) level at birth (day 1) clearly differed from that of pre- (day 42) and postweaning (day 70) animals with the increased microbial diversity (Fig. 3e), consistent with established findings from calves and lambs [35, 36]. Importantly, specific core microbiota for each distinct GIT region such as the rumen (*Lactobacillus* spp.), small intestine (*Lactobacillus* spp.), and large intestine (*Bacteroides* spp.) showed the most noticeable and significant decrease at days 42 and 70 when compared to day 1 (Fig. 3f), confirming the previous observation in the small and large intestine of neonatal calves [5, 37], and further revealing the heterogeneous colonization in each GIT segment. However, this result is inconsistent with previous findings from the neonatal calf rumen, which was shown to be dominated by the genera *Prevotella* spp., *Ruminococcus* spp., and *Streptococcus* spp. [6, 35, 38]. This difference is likely reflected by the effects of maternal vertical transmission (i.e., colostrum, skin, and vagina) as well as the environmental microbiota (i.e., feces) that presumably affect stochastic microbiota in the rumen [2, 39].

As expected, the prevalence of the identified microbial genera significantly varied during early life. The relative abundances of *Prevotella* spp. and *Fibrobacter* spp. increased in the rumen, while *Romboutsia* spp. and *Clostridium* spp. increased in small intestine (jejunum and ileum), and *Alistipes* spp., *Oscillibacter* spp., *Bacillus* spp., *Ruminococcus* spp., and *Flavonifractor* spp. increased in large intestine (cecum and colon, Fig. 3f). *Prevotella* have the capability to utilize starches, simple sugars, and other non-cellulosic polysaccharides as energy [40], whereas the genus *Fibrobacter* are believed one of the major degraders of cellulosic plant biomass frequently found in the adult rumen [41]. The genera *Alistipes*, *Butyrivibrio*, and *Flavonifractor* are important butyrate producers in gut [42]. In addition, *Oscillibacter* spp. has previously been observed with high heritability from preweaning to postweaning in the large intestine of bovine [4]. *Clostridium* spp. could possibly strengthen the mucosal barrier by increasing the thickness of the inner mucus layer [43]. Overall, our results indicated a determined role of each GIT regions in microbial succession, and that different microbial communities likely contribute to carbohydrate fermentation and imply a possibly distinct metabolic profile in the rumen and large intestine.

### The altruism type-driven GIT microbiota assembles from birth to postweaning

**Fig. 4:**
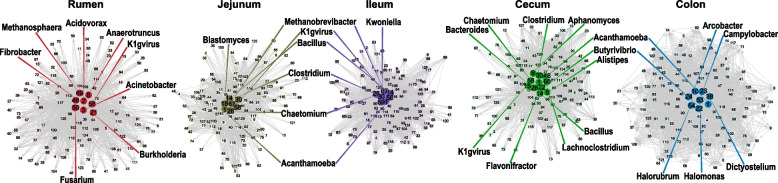
Microbial *Z*_al_-based altruism network in the GIT of sika deer. The altruism network at the genus level within the gut microbiota in rumen, jejunum, ileum, cecum, and colon. In each network, hub microbes are highlighted in dark circles. These hub microbes, expressed as beneficiaries in altruism networks, are compared with other microbes from each network type, called altruists

To further understand the mechanisms behind the microbial community shifts observed in our data, we integrated behavioral eco-evolutionary theory to reflect the possible types of microbe-microbe interactions, including mutualism, antagonism, aggression, and altruism [44]. The results showed that an altruism network was the dominant interaction type in rumen, jejunum, ileum, cecum, and colon (Figs. 4 and S7), suggesting that a cooperative manner is important for GIT microbial community assembly at early life. We then identified the so-called hub microbes, which usually display relatively higher degree and closeness centrality scores than other populations and can promote the balance of microbial interactions within the network and maintain network function [44]. The results suggested fibrolytic *Fibrobacter* spp. in the rumen, *Bacillus* spp. and *Clostridium* spp. in the small intestine and butyrate-producers *Alistipe*s spp., and *Butyrivibrio* spp. in the large intestine are all hub populations (Fig. 4**)**. Our observations of significantly increased hub taxa in each GIT regions could be used to speculate that the resultant metabolic profile is likely a factor that affects microbial community assembly. However, we also found taxa with lower abundance including bacteria, archaea, protozoa, fungi, and phage that were also identified as hub microbes, possibly contributing to the overall metabolic capacity and shift of GIT microbiota from birth to postweaning (Supplementary Discussion). Our findings support previous study that low-abundance microbes may contain a pool of genes that can contribute to the complete metabolic potential of the community if they are highly active, enhance or trigger the metabolic activity of more dominant members, or contain enzymes needed for complex metabolic processes that are not found in the dominant members [45].

### Different tendency of metabolic profile and metabolites in GIT development

**Fig. 5:**
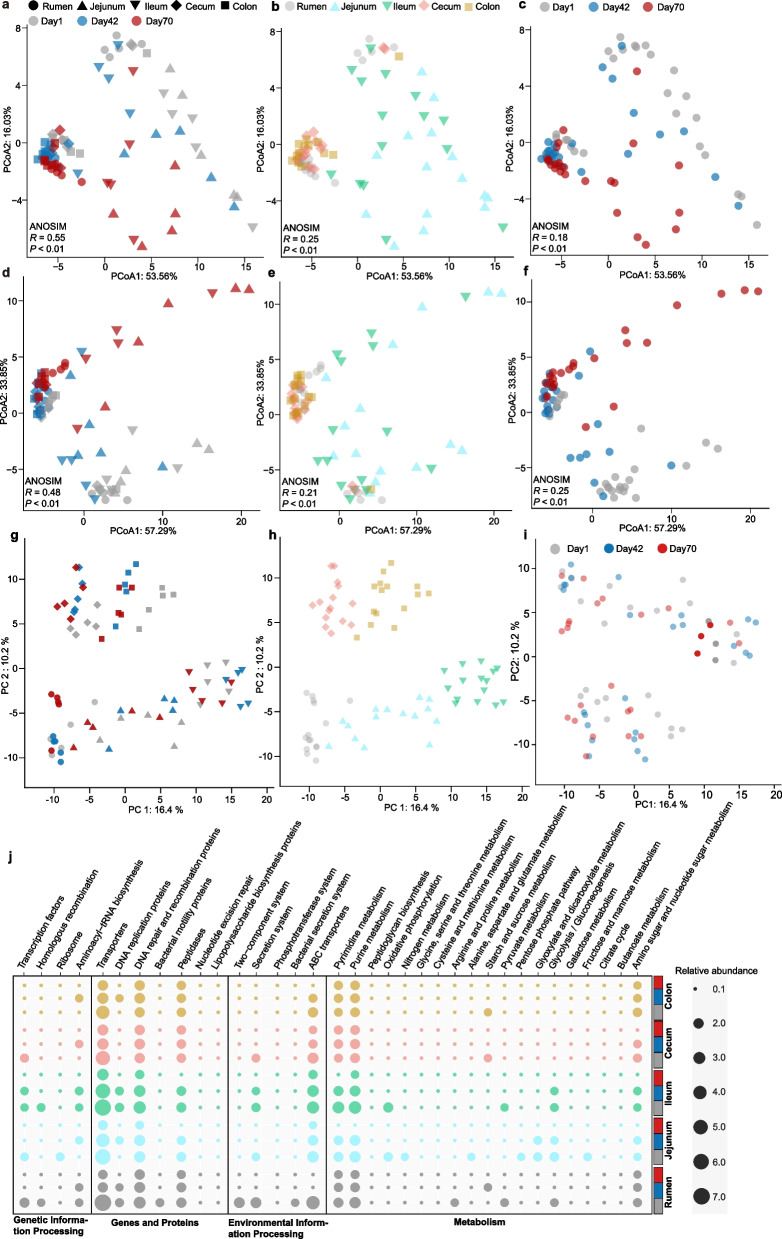
Metabolic signature in GIT regions of sika deer from birth to postweaning. PCoA of GIT metabolic profiles at KO (**a**, **b**, **c**) and KEGG level 3 (**d**, **e**, **f**) based on Bray-Curtis dissimilarity. ANOSIM analysis was used for statistical testing of group similarities. The proportion of variation explained for each axis is given after colon. **g**, **h**, and **i** PCA of metabolome in five GIT regions. The samples from GIT regions among the three time points indicated as different shapes (rumen: circle, jejunum: triangle, ileum: inverted triangle, cecum: rhombus, and colon: square) and filling color (day 1: gray, day 42: blue, day 70: red) in **a**, **d**, and **g**. **j** The significantly changed pathways. The circle sizes indicate the relative abundance of each pathway and were colored by GIT regions (rumen: gray, jejunum: light blue, ileum: light green, cecum: pink, and colon: yellow). The significances were determined a Benjamini-Hochberg-adjusted *P*-value < 0.05 using relative abundances of KEEG level 3. From bottom to top: rumen, jejunum, ileum, cecum, and colon including three time points (day 1: gray, day 42: blue, day 70: red). Full results were supplied in Table S5

To explore the underlying metabolic profiles across the GIT, we characterized the Kyoto Encyclopedia of Genes and Genomes (KEGG) profiles of predicted genes from our metagenomic inventories. PCoA results showed that the sika deer GIT microbiome functional profile at KO (Fig. 5a–c), KEGG level 1 (Fig. S8a–c), KEGG level 2 (Fig. S8d–f), and KEGG level 3 (Fig. 5d–f) and carbohydrate active enzyme (CAZyme) profile (Fig. S8g–i) of day 1 differed from that of days 42 and 70, with a much clearer separation in the rumen (Fig. S9a) and large intestine (Fig. S9b). Moreover, we observed that the microbial functions (KOs and KEGG pathway) in the small intestine differed from that of the rumen and large intestine across all three time periods, while the diversity of CAZyme profile significantly increased at day 42 comparing that at day 1 (Fig. S10). This is agreement with the compositional plasticity and confirmed the presence of the fermentative and enzymatic activities (xylanase and amylase) in rumen and cecum [46], suggesting that microbiome in both the rumen and large intestine has established the ability to catalyze fiber plants soon after weaning. However, there was an observed discordance for the changed patterns between metabolites and microbiome structure in the GIT. PCA results showed the metabolites disordered according to rumen, small intestine, and large intestine (Fig. 5g–h) but with limited time-dependent effects (Fig. 5i). This observation is further supported by the metabolite variants contributing to the distinction among the three time periods (Fig. S11). These results indicated the metabolic profile is relatively constrained in each GIT region. A recent study demonstrated that stochastic colonization in early life has long-lasting impacts on the development of rumen microbiome [3]. Together, our results propose the hypothesis that the metabolome profiles in each GIT region may have been determined and constrained by the firstly colonized microbiomes during early life, highlighting the importance combining microbial function and metabolites to understand the GIT metabolism.

Further comparison of the KEGG pathways at level 1 showed significant differences in the GIT among the three time points (Table S4). A total of 106 (rumen) and 59 (colon) significantly changed pathways at KEGG level 3 were observed with decreased relative abundances among the three time points (Fig. 5j and Table S5), such as the pathways of pyruvate metabolism, butanoate metabolism, and citrate cycle within the category of metabolism. Accordingly, the concentrations of VFA including acetate, propionate, and butyrate also significantly increased in rumen and large intestine (Fig. S12). These results solidify that the metabolic change in rumen and colon is likely important contributors to host metabolism and infer that the metabolic adaptation in both regions is likely a result from a reduction of metabolic specificity and an increase of metabolic diversity from birth to postweaning.

### A characteristic of carbohydrate metabolism in the rumen and amino acids metabolism in the colon

**Fig. 6:**
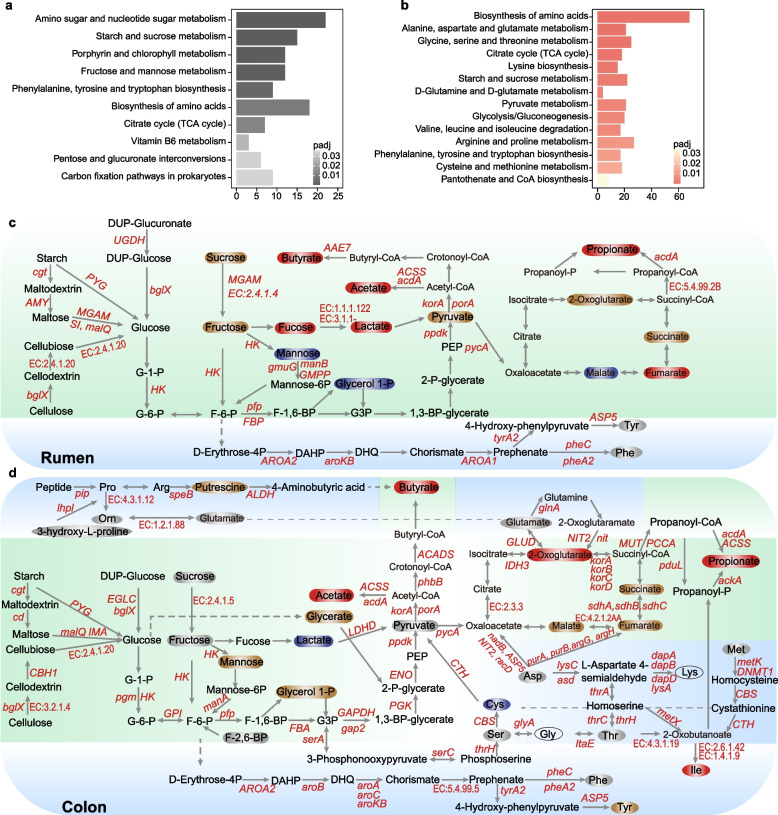
Integrative metabolic view in the rumen and colon of sika deer between birth and postweaning. Histogram showing the significantly enriched pathways in rumen (**a**) and colon (**b**). Pathway enrichment analysis was performed by a one-side Fisher test based on the significantly increased KOs (full results were supplied in Table S6). The significance of KOs was determined by a Benjamini-Hochberg-adjusted *P*-value < 0.05 based on the relative abundance between day 70 vs day 1. The x-axis represents the gene sets in each pathway. The graphs of the metabolic pathways of carbohydrate and amino acids in rumen (**c**) and colon (**d**). The light green and light blue backgrounds represent the metabolism of carbohydrate and amino acids, respectively. The rounded rectangle and ellipse indicate metabolites associated with carbohydrate and amino acids, respectively. Red- and brown-rounded rectangle/ellipse indicate the significantly increased/increased, while blue and gray rectangle/ellipse indicate the significantly decreased/decreased. The significant increase of enzyme codes and gene names between day 70 vs day 1 in the pathways was indicated as red text. P, phosphate; PEP, phosphoenolpyruvate; G3P, glycerol 3-phosphate; Tyr, tyrosine; Phe, phenylalanine; Ser, serine; Cys, cysteine; Thr, threonine; Ile, isoleucine; Asp, aspartate; Met, methionine; Lys, lysine; Gly, glycine; Pro, proline; and Arg, arginine

To gain a deeper insight of the metabolic shift from birth (day 1) to postweaning (day 70), we subsequently reconstructed metabolic pathways using the significantly increased KOs and metabolites in rumen and colon. The results showed that both starch and sucrose metabolism and citrate cycle pathways within the category of carbohydrate metabolism were significantly enriched (Fig. 6a), indicating the two metabolic profiles played core roles in foregut and hindgut of juvenile ruminant growth. Moreover, the fructose and mannose metabolism pathway were also specifically enriched in the rumen, where the concentrations of sucrose, fructose, and lactate in rumen increased at day 70 relative to that at day 1, but mannose and glycerol 1-P decreased (Figs. S11 and S13). These results suggested an important role of mannose fermentation via hemicellulose hydrolysis in the rumen after weaning.

In addition, we found that the pathways of amino acids metabolism (alanine, aspartate, glutamate, glycine, serine, threonine, valine, leucine, isoleucine, arginine, proline, cysteine, and methionine) were specifically enriched in the colon (Fig. 6b). Concurringly, the concentrations of aspartate, glutamate, serine, threonine, cysteine, and methionine also decreased from day 1 to day 70 (Figs. 6b, S11, and S14), inferring their metabolism and/or absorption from the host. In vitro studies have shown that propionate is produced mainly from aspartate, alanine, threonine, and methionine fermentation, whereas butyrate is a major product from the fermentation of glutamate, lysine, cysteine, serine, and methionine [47]. *Oscillospira* species, identified to increase at days 42 and 70 in large intestine, are known to produce butyrate via fermentation of host glycans (such as sialic acids and glucuronic acid) and are associated with reduced incidence of inflammatory disease [48]. Collectively, our results suggest that the metabolism of amino acids was important for VFA production in the colon of sika deer after weaning, and potentially these identified amino acids may provide a source to sustain the healthy gut of juvenile ruminants at early life stage.

In agreement with our data that suggests increases in rumen hydrolysis and fermentation at day 70 (compared to day 1), the concentration of pyruvate increased, whereas in contrast it was observed to decrease in the colon (Fig. 6c–d). The succinate pathway presented in *Ruminococcus* spp. and *Alistipes* spp., and the propanediol pathway which has been demonstrated in *Flavonifractor* spp. and *Blautia* spp., is all known for the formation of propionate. Accordingly, the abundance of 4 (rumen) and 10 (colon) metabolic genes involving in production of propionate and butyrate from pyruvate significantly increased (Fig. 6c–d). Meanwhile, the abundance of 10 genes linked to the citrate cycle also significantly increased in colon at day 70 in comparison with that at day 1. These results suggested that the enhanced production of pyruvate in rumen, and the utilization of pyruvate in colon, is likely responsible for the increased production of VFAs. Together, these results suggest distinct metabolic adaptation within the rumen and colon during early life, with important carbohydrate metabolisms such as mannose in rumen and amino acids in colon, respectively.

### Transcriptomic analysis of GIT epithelium of sika deer after birth

**Fig. 7:**
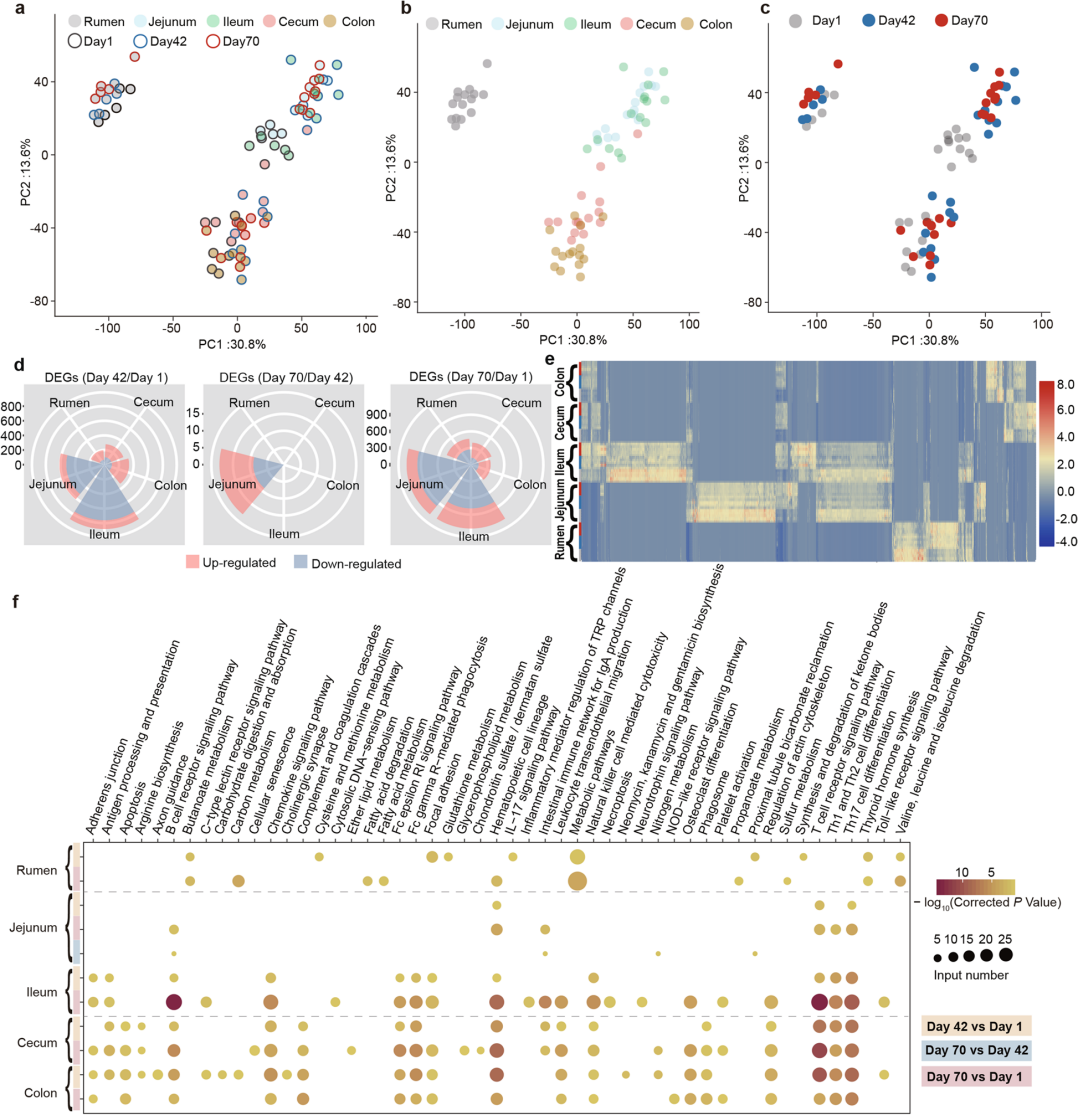
Comparative analysis of GIT epithelial function at early life development stages of sika deer. PCA of all expressed genes in 5 rumen, jejunum, ileum, cecum, and colon among the three time points (**a**–**c**). PCA vector separates samples into GIT regions using fill color of circle (rumen: gray, jejunum: light blue, ileum: light green, cecum: pink, and colon: yellow) and age groups using outer line color (day 1: gray, day 42: blue, day 70: red) **a** and **b**. PCA vector separates samples into age groups using different filling color (day 1: gray, day 42: blue, day 70: red) in **c**. **d** Rose diagrams showing the numbers of DEGs in the five GIT regions. Pink and blue represent the up- and downregulated DEGs, respectively. The DEGs were determined by the fold change ≥ 2 and a Benjamini-Hochberg-adjusted *P*-value < 0.05. **e** Heat map showing the distribution of DEGs in rumen, jejunum, ileum, cecum, and colon (from bottom to top). Each row represents one sample, and each column represents one gene. Individuals are colored (blue to red) to indicate expression level (low to high). **f** Representative pathways enriched in upregulated DEGs based on functional enrichment analysis in five GIT regions. The vertical axis represents the pathway categories, and the horizontal axis shows the enrichment factor. The circle size represents the number of gene sets. The bigger the point size, the more genes in the pathway. The circle color indicates the logarithm of *P*-values calculated as the fraction of permutation values. The color legend at the left of heat map indicates the different comparisons

To further explore the adaption and functional changes of the GIT epithelium during growth, we conducted RNA-seq-based transcriptomics, and obtained a total of 547.96 Gb reads, with an average of 84.30 ± 0.20% mapping rate to the high-quality sika deer reference genome (unpublished, Contig N50 = 9.5 M, Table S7). An average of 9811 ± 41 expressed genes (FPKM ≥ 1) was identified in each sample (Table S8). PCA results showed that the gene expression of GIT epithelium at day 1 differed from that at days 42 and 70 (Fig. S15), which is consistent with the previously observed findings in GIT regions of neonatal calves, goat, and sheep [6, 7, 12, 49], indicating an ontogenic event of GIT tissue function [10]. Obviously, we observed that rumen, small intestine, and large intestine clustered separately (Fig. 7a–c), which showed an overall consistency within the shift of GIT metabolites (Fig. 5g–h). It is reported that the introduction of different solid diets differently affected rumen epithelial morphology [50], which is also associated with the microbiome-driven metabolites, acetate, and butyrate, affecting the growth-associated signaling pathway [7]. These findings suggested a potential role of metabolite-driven transcriptional regulation of juvenile ruminant GIT function after birth.

The results of identified DEGs in each GIT region showed the significant shift occurred between day 42 and day 1 (Fig. 7d), indicating that the period from birth to preweaning is a hinge of GIT epithelium functional development. Moreover, the DEGs in varying GIT regions among three time points displayed tissue-specific transcriptional patterns (Fig. 7e), with both cecum and colon (Fig. S16) sharing more up- and downregulated DEGs than observed in both the jejunum and ileum (Fig. S17). This discrepancy in region-specific DEGs suggested a distinct function development of intestinal regions, with a very dynamic shift in the small intestine and a lesser change in the large intestine.

Because of the regional transcriptome differences, we then examined the changes associated with metabolic pathways based on these identified DEGs in the rumen, small intestine, and large intestine, respectively. Within the rumen, a total of 90 genes specially and significantly enriched in the pathways including butanoate metabolism (*Echs1*, *Hmgcs2*, *Acads*, *Bdh1*), valine, leucine, and isoleucine degradation in rumen (*Hmgcs2*, *Acads*, *Echs1*, *Pcca*, *Acaa2*, *Acadsb*; Figs. 7f, S18a, c and Table S9), as well as 61 genes, were downregulated from birth to postweaning (Figs. S18b, d). *Hmgcs2* is a rate-limiting gene in ruminal ketone body synthesis, which was also upregulated in rumen epithelium of developing sheep [50]. The stratified squamous epithelium structure of the rumen is the domain site for VFA absorption and utilization, which also stimulated the development of rumen papillae [51]. Consistently, butanoate metabolism was shown to be the most significantly enriched signal pathway in ruminal tissue with alfalfa hay or concentrate starter introduction to lamb [50]. Moreover, the BCAAs could provide substrates to citric acid cycle by *Acads*, *Echs1*, and *Pcca*, generating energy-containing compounds, including NADH, FADH, and ATP [52]. Thus, our results indicated a key role of butyrate and BCAAs metabolism in the autogenetic development of rumen wall morphology and function from birth to postweaning. We also revealed that the upregulated DEGs in the rumen (day 70 vs day 1) were specially enriched in pathways of propanoate metabolism, fatty acid metabolism, and degradation. Previous studies demonstrated that the catabolism of BCAAs regulates the trans-endothelial flux of fatty acids affecting whole-body metabolic homeostasis [53]. Our results support well-established dogma that rumen metabolism is a likely reasonable source of fatty acids for the host and further reveals a cross-regulatory link between the catabolism of BCAAs and FAs.

In the small and large intestine, we observed that B-cell receptor signaling pathway, T-cell receptor signaling pathway, Th17 cell differentiation, Th1 and Th2 cell differentiations, and hematopoietic cell lineages were significantly enriched with animal growth, particular to the comparison between day 70 vs day 1 in the ileum, cecum, and colon (Figs. 7f, S16 c–d, and S17c–d, Supplementary Discussion). This is consistent with previous finding in calves [49] and goats [12], highlighting the role of complex and dense innate and adaptive immune response influencing small and large intestine development from birth to postweaning. In addition to noted changed in immune response of GIT, we showed that the necroptosis (*Traf5*, *Stat1*, *Ifng*, *Zbp1*, *Jak3*, *Fas*, *Il1b*) and apoptosis (*Csf2rb*, *Fos*, *Gadd45b*, *Bcl2a1*, *Parp3*, *Ctsw*) were specially enriched in the ileum (Figs. 7f and S17d) and large intestine (Figs. 7f and S16c—d), respectively. Apoptosis is a process relying on caspase activation, while necroptosis occurs via programmed cell necrosis, negatively regulated by caspases, and depending on the kinase activity of receptor-interacting proteins. The expression level of *Fos* is possibly related to the proliferation and apoptosis of the intestinal epithelial cells [54]. The *Gadd45b*-regulated TGF-β signaling pathway is involved in enhancing epithelial restitution in colon [55]. Indeed, recent data suggested that the expression of the antiapoptotic gene *Bcl2* in colonic crypts protects these cells from spontaneous apoptosis, which are direct targets of the NF-κB-signaling cascade and act as pro-survival factors [56]. Together, these findings implicated the differences in the regulation of cell death between the ileum and colon from birth to postweaning.

In addition, the pathways including arginine biosynthesis (*Nos2*, *Ass1*), phagosome (*Tap1*, *Ncf2*, *Ncf1*, *Coro1a*, *Itgam*, *C3*), and complement and coagulation cascades (*Cfd*, *Masp1*, *Itgam*, *C3*, *C6*) were specially identified in the large intestine (Figs.7f and S16 c–d). *Ass1* encodes the rate-limiting enzyme leading to de novo synthesis of arginine, an important amino acid for the growth of intestinal epithelial cells. The upregulation of *Ass1* contributes to epithelial proliferation necessary to be sustained during regeneration [57]. *Nos2* is a homodimeric gene converting arginine into nitric oxide, which exerts a central role in epithelial barrier integrity and mucosal homeostasis of intestine [58]. Nitric oxide is important moderators in the control and escape of infectious pathogens in T- and B-cell differentiation [59]. These results indicated the arginine synthesis and metabolism are highly activated in the colon of sika deer. C3, CR3 (ITGAM), and CFD (complement factor D) were key components in the activation of the complement system, which is a functional bridge between innate and adaptive immune responses that allows an integrated host defense to pathogenic challenges [60]. Moreover, previous study demonstrated that complement induces the expression of intestinal *Nos2* [61]. Together, these results suggested a regulator role of arginine metabolism in affecting immune response of colon at early life.

## Conclusion

In this study, we applied multiple omics approaches across contrasting sites across the host-microbiome axis to systemically understand the association between the GIT microbiome, metabolites, epithelial function, and host metabolic traits. We demonstrated the fatty acid composition in *LL* significantly changed with animal growth, which is consistently reflected to urinary metabolites. Further transcriptome and metabolome analysis identified the key amino acids (tryptophan and BCAAs) in liver metabolic adaptation processes with a dramatic shift after weaning, while the fatty acid β-oxidation-induced cholesterol metabolism is likely enhanced in liver after weaning. Our metagenomic and metabolic data across spatial GIT sites are as follows: (1) documented the diverse microbiota rapidly colonized in GIT with regional effects after birth, (2) revealed a constrained effect within each GIT region on metabolites profiles and identified the specially changed metabolites, and (3) demonstrated distinct levels of metabolic adaptation in the rumen and colon during early life. Finally, GIT epithelium transcriptome data demonstrated the importance of fatty acids in the rumen, and the immune response influencing functional shifts within the small and large intestine, and indicated the potentially role of necroptosis and apoptosis in coordinating the development of ileum and colon from birth to postweaning, respectively. In summary, our work reported here provides evidence that designed interventions that target GIT ecology niches and their specific relationships with metabolic adaptation of their host at early life stages can likely contribute to improving juvenile ruminant health through regime strategies.

## Methods

### Animals and sample collection

A total of 15 healthy juvenile sika deer from previous studies were used [62]. Briefly, fifteen pairs of juvenile sika deer were kept with their dams in three pens, with 5 pairs of juvenile sika deer and dams in each pen. All sika deer were obtained and raised at the research farm of the Institute of Special Animal and Plant Sciences, Chinese Academy of Agricultural Sciences. The juvenile sika deer suckled their young before weaning (day 60) and also had access to the concentrate (50%) and corn stover silage (50%, dry matter base). After weaning at day 60, five young animals were separated from their dams, were maintained in an individual outdoor pen without any bedding materials, and were offered with corn silage and concentrate diets. All animals had free access to clean water during this study.

To decrease effect of sampling time points, each five juvenile sika deer after morning feeding (3 h) from days 1, 42, and 70 was slaughtered at each time point, respectively. Blood, urine, and whole *longissimus lumborum* (*LL*) samples were collected from the jugular vein, bladder, and right side of each sika deer, respectively. The GIT regions including rumen, jejunum, ileum, cecum, and colon were tied off using cotton rope, and then the content samples from each region were collected. The GIT epithelium of each region (middle location) and liver tissue were washed three times with cold sterile phosphate-buffered saline (PBS, pH = 7.0) and transferred to RNAlater preservation solution (Thermo Fisher Scientific, Waltham, USA). Afterward, the collected samples were snap-frozen in liquid nitrogen and then stored at −80 °C for further analysis. All animal-specific procedures were approved and authorized by the Animal Ethics Committee of Jilin Agricultural University and the Chinese Academy of Agricultural Sciences Animal Care and Use Committee.

### Measuring fatty acid (FA) and amino acid (AA) composition in LL

The FA composition of *LL* samples was determined after extracting total lipids as previously described [63]. Gas chromatography conducted with (HP 6890, Agilent Technologies, CA, USA) a DB-23 capillary column (60 m × 0.25 mm × 0.25 μm) (Agilent Technologies, CA, USA) was used to determine FAs composition according the previous method [64]. For determining the composition of AA, protein hydrolysis, and derivatization and identification of hydrolyzed amino acids were analyzed using a HPLC (Agilent Technologies, CA, USA) and detected using a scanning fluorescence detector according to the previous method [65].

### DNA and RNA extraction, shotgun sequencing, and metabolome analysis

DNA extraction was performed as previously described [66]. Briefly, the genomic DNA was extracted from GIT content samples (~200 mg per sample) following the protocol based on repeated bead beating using a mini-bead beater (Biospec Products, Bartlesville, USA). For RNA isolation, the GIT tissue and liver samples were ground into powder in liquid nitrogen, which were used for total RNA extraction using mirVana™ miRNA Isolation Kit (Ambion, CA, USA) following the manufacturer’s instructions. RNA samples with an integrity number (RIN) greater than 7.0 were used for library construction. Total genomic DNA (1.0 μg) was used to construct pair-ended libraries using the TruSeq DNA PCR-Free Library Preparation Kit (Illumina, CA, USA) with an insert size of 350 bp. One microgram of RNA from GIT tissue or liver samples was used to construct the RNA-Seq library using NEBNext® Ultra™ RNA Library Prep Kit (Illumina, CA, USA). Each library was quantified using a Qubit 2.0 Fluorometer (Invitrogen, CA, USA) and then sequenced on an Illumina HiSeq 4000 platform (150 bp paired-end sequencing).

### Metagenome and transcriptome bioinformatic analysis

For the metagenomic analysis, the obtained raw sequences were first processed using Trimmomatic (version 0.36) [67] for quality control to remove adapters, low-quality bases (quality scores < 25), and reads with length < 50 bp. BWA-MEM (version 0.7.17) [68] was used to remove the host-associated reads by aligning to the reference genome of sika deer (unpublished). The filtered reads from each sample were de novo assembled using MEGAHIT (version 1.1.1) [69] with the option of min-contig-len 500. Open reading frames (ORFs) from each sample were predicted with GeneMarkS (version 2.7) [70] with the parameter: gmhmmp-m MetaGeneMark_v1.mod. CD-HIT (version 4.6.6) [71] was used to construct a non-redundant gene catalog with 95% cutoff sequencing identity and 90% coverage.

Kraken 2 [72] was used to identify the taxonomic information based on the nonredundant gene catalog. For functional annotation, the amino acid sequences translated from the integrated gene catalog were aligned against protein or domain sequences in the Kyoto Encyclopedia of Genes and Genomes (KEGG) databases (version 90.0) [73] using DIAMOND (version 0.8.24.86) [74] with a cutoff *e*-value < 1e-5, which assigns proteins to KEGG orthology (KO) identifiers based on the highest-scoring annotated hit. The prediction of CAZymes was conducted by using HMMER [75] (v.3.2.1) to match protein sequences to entries in the hidden Markov model (HMM) libraries of CAZyme families downloaded from the CAZy database [76]. The high-quality reads from each sample were aligned against the gene catalogs using BWA-MEM [68] (version 0.7.17), and abundance profiles of genes (alignment length ≥ 50 bp and sequence identity > 95%) were calculated in transcripts per million (TPM), with corrections for variations in gene length and mapped reads per sample. The relative abundances of taxa, KO, and CAZymes were calculated from the abundances of annotated genes. PCoA based on the Bray-Curtis dissimilarity matrices was applied to visualize the variation of taxonomic composition and functional features. Analysis of similarities (ANOSIM) was used to indicate the group similarity of differences.

For the transcriptome analysis, Trimmomatic (version 0.36) [67] was used to remove the low quality and adapter sequence. After that, the remaining reads were aligned to sika deer reference genome using HISAT2 (version 2.2.0) [77]. The expression profiles of mRNAs in each sample were calculated by normalizing reads number to fragments per kilobase million (FPKM) using StringTie (version 2.1.3b) [78]. The differently expressed genes (DEGs) were identified using edgeR [79]. The significantly DEGs were determined by the fold change ≥ 2 and false discovery rate (FDR) < 0.05, and the FDR was calculated based on Benjamini and Hochberg correction. The KEGG enrichment analyses of DEGs were performed using KOBAS (version 3.0) [80]. PCA was applied to reveal the gene expression.

### Identifying metabolites in plasma, urine, liver, and GIT contents

Gas chromatography-mass spectrometry (GC-MS) was applied to characterize the metabolites in the plasma, urine, liver, and rumen contents using the reported method [62]. The obtained metabolites with a criterion of similarity greater than 300 from the LECO/Fiehn Metabolomics library were retained for the further analysis. By combing the previously obtained metabolites in jejunum, ileum, cecum, and colon [62, 81], these data were firstly used to log transformation and pareto scaling using MetaboAnalyst 3.0 platform [82] and were used to conduct the multivariate analysis including the PCA and PLS-DA analysis. The significantly changed metabolites were identified using variable VIP values that exceeded 1.0 and *P* < 0.05, significance analysis of microarray (SAM) based on F-statistics, and one-way analysis of variance (ANOVA).

### Networks of GIT microbiome

To explore the changed association of GIT microbiome, the microbial taxa was used to reconstruct interaction network based on the microbial behavioral network model [44]. According to this model, four mathematical descriptors (mutualism, antagonism, aggression, and altruism) were counted to showed types of microbial interactions using a network, which was calculated as *Z*_mu_, *Z*_an_, *Z*_ag_, and *Z*_al_, respectively. Then we described the properties of a network by calculating six centrality indices, including connectivity (Con), closeness (C(u)), betweenness(B(u)), eccentricity (E(u)), eigenvector (G(u)), and PageRank (P(u)). The so-called hub or keystone microbes were also identified by calculating the degree and closeness of each taxa in network by the “igraph” R package. The network was visualized using Cytoscape (version 3.8.2) [83].

### Statistical analysis

ANOVA was used to determine the difference of FA, AA, microbial taxa, and functional profiles among the three groups. The *P*-values were corrected using the FDR and the Benjamini-Hochberg correction, and a *P*-value < 0.05 was regarded as statistically significant. If the ANOVA comparison indicated significance, we also applied the Wilcoxon rank-sum (WRS) test to determine the significance between pairs of groups: day 1 vs day 42, day 42 vs day 70, and day 1 vs day 70.

## Supplementary Information

**Additional file 1: Figure S1.** Fatty acid profiles in *longissimus lumborum* (*LL*) of sika deer from birth to postweaning. **Figure S2.** Amino acid profiles in *longissimus lumborum* (*LL*) of sika deer from birth to postweaning. **Figure S3.** Variational characteristics of serum metabolites in sika deer from birth to postweaning. **Figure S4.** Urine metabolic profile of sika deer from birth to postweaning. **Figure S5.** Transcript and metabolic shifts in the liver of sika deer from birth to postweaning. **Figure S6.** Variation of GIT microbial community composition from birth to postweaning in sika deer. **Figure S7.** Microbial interaction networks in the rumen, jejunum, ileum, cecum and colon microbiome of sika deer. Figure S8. Changes in the GIT microbial metabolic profiles in sika deer from birth to postweaning. **Figure S9.** Changes in KEGG level 3 and CAZy annotations in five GIT regions from birth to postweaning. **Figure S10.** Comparison of diversity indices for functional annotations generated from the GIT of sika deer over early life development. **Figure S11.** Heatmap showing the global change of GIT metabolites from birth to postweaning in sika deer. **Figure S12.** Comparison of VFA concentrations measured from the rumen (a) and colon (b) of sika deer during early life development stages. **Figure S13.** Metabolic profile in the rumen content of sika deer from birth to postweaning. **Figure S14.** Significantly changed metabolites detected in the jejunum (a), ileum (b), cecum (c) and colon (d). **Figure S15.** PCA of all expressed genes in host tissue sampled from the rumen (a), jejunum (b), ileum (c), cecum (d) and colon (e). **Figure S16.** Changes in the cecum and colon epithelium transcriptome from birth to postweaning in sika deer. **Figure S17.** Changes in the jejunum and ileum epithelium transcriptome from birth to postweaning in sika deer. **Figure S18.** Transcriptomic differences observed in the rumen epithelium in sika deer from birth to postweaning.

**Additional file 2: Table S1.** Summary of RNA-seq data from liver tissue of sika deer among the three stages. **Table S2.** Enrichment analysis at KEGG level 3 of up regulated DEGs in liver among the three stages.

**Additional file 3: Table S3.** Sequencing data of the 75 GIT samples of sika deer among the three stages. **Table S4.** The significantly changed metabolic pathways at KEGG level 1 in five GIT regions among the three time points. **Table S5.** The significantly changed metabolic pathways at KEGG level 3 in the rumen and colon among the three time points. **Table S6.** Enrichment analysis of the increased KOs in the rumen and colon microbiota between the comparison of Day 70 vs Day 1.

**Additional file 4: Table S7.** Summary of genome assembly of Sika deer. **Table S8.** Summary of RNA-seq data from 75 GIT epithelium samples of sika deer. **Table S9.** Enrichment analysis at KEGG level 3 of up regulated DEGs in rumen epithelium.

**Additional file 5.** Supplementary Discussion.

## Data Availability

Raw sequence reads for all samples are available under NCBI project PRJNA779578 (metagenome data) and PRJNA777048 (RNA-seq data).
