# Supplementary material for: Integrated multi-omics of the gastrointestinal microbiome and ruminant host reveals metabolic adaptation underlying early life development
Source: Microbiome. 2022 Dec 12;10:222. doi: 10.1186/s40168-022-01396-8 (PMC9743514; doi:10.1186/s40168-022-01396-8)
Supplement: Supplementary file 2 — Additional file 1: Figure S1. Fatty acid profiles in longissimus lumborum (LL) of sika deer from birth to postweaning. Figure S2. Amino acid profiles in longissimus lumborum (LL) of sika deer from birth to postweaning. Figure S3. Variational characteristics of serum metabolites in sika deer from birth to postweaning. Figure S4. Urine metabolic profile of sika deer from birth to postweaning. Figure S5. Transcript and metabolic shifts in the liver of sika deer from birth to postweaning. Figure S6. Variation of GIT microbial community composition from birth to postweaning in sika deer. Figure S7. Microbial interaction networks in the rumen, jejunum, ileum, cecum and colon microbiome of sika deer. Figure S8. Changes in the GIT microbial metabolic profiles in sika deer from birth to postweaning. Figure S9. Changes in KEGG level 3 and CAZy annotations in five GIT regions from birth to postweaning. Figure S10. Comparison of diversity indices for functional annotations generated from the GIT of sika deer over early life development. Figure S11. Heatmap showing the global change of GIT metabolites from birth to postweaning in sika deer. Figure S12. Comparison of VFA concentrations measured from the rumen (a) and colon (b) of sika deer during early life development stages. Figure S13. Metabolic profile in the rumen content of sika deer from birth to postweaning. Figure S14. Significantly changed metabolites detected in the jejunum (a), ileum (b), cecum (c) and colon (d). Figure S15. PCA of all expressed genes in host tissue sampled from the rumen (a), jejunum (b), ileum (c), cecum (d) and colon (e). Figure S16. Changes in the cecum and colon epithelium transcriptome from birth to postweaning in sika deer. Figure S17. Changes in the jejunum and ileum epithelium transcriptome from birth to postweaning in sika deer. Figure S18. Transcriptomic differences observed in the rumen epithelium in sika deer from birth to postweaning. [file 40168_2022_1396_MOESM1_ESM.docx]

**Supplementary Figures**

**
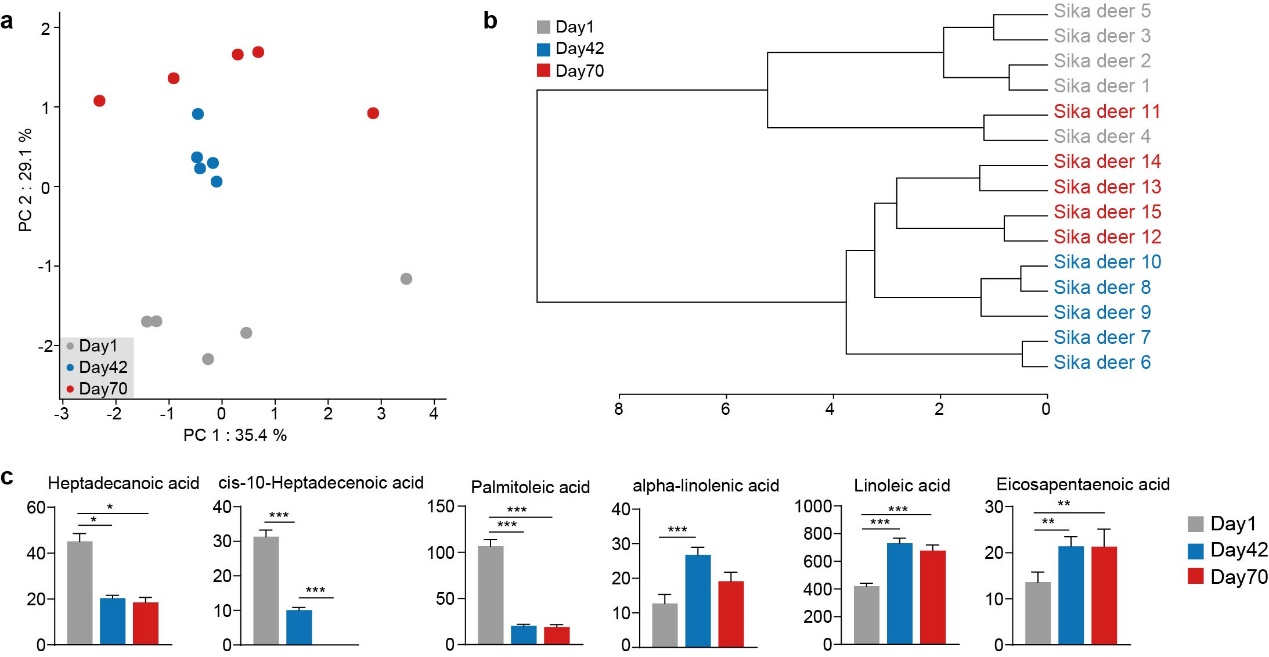
**

**Figure S1. Fatty acid profiles in *longissimus lumborum* (*LL*) of sika deer from birth to postweaning**. **(a)** PCA and (**b**) hierarchical clustering of fatty caid composition in *LL*. Samples are colored by age groups (Day 1: gray, Day 42: blue, and Day 70: red). (**c**) Box plots showing 6 fatty acids significantly changed during early growth period (Concentration=mg/kg). Benjamini-Hochberg-adjusted *P*-values were determined by ANOVA. Bar and whiskers represent the mean ± s.d. *, **, and *** indicate the Benjamini-Hochberg-adjusted *P*-value <0.05, <0.01 and <0.001, respectively.


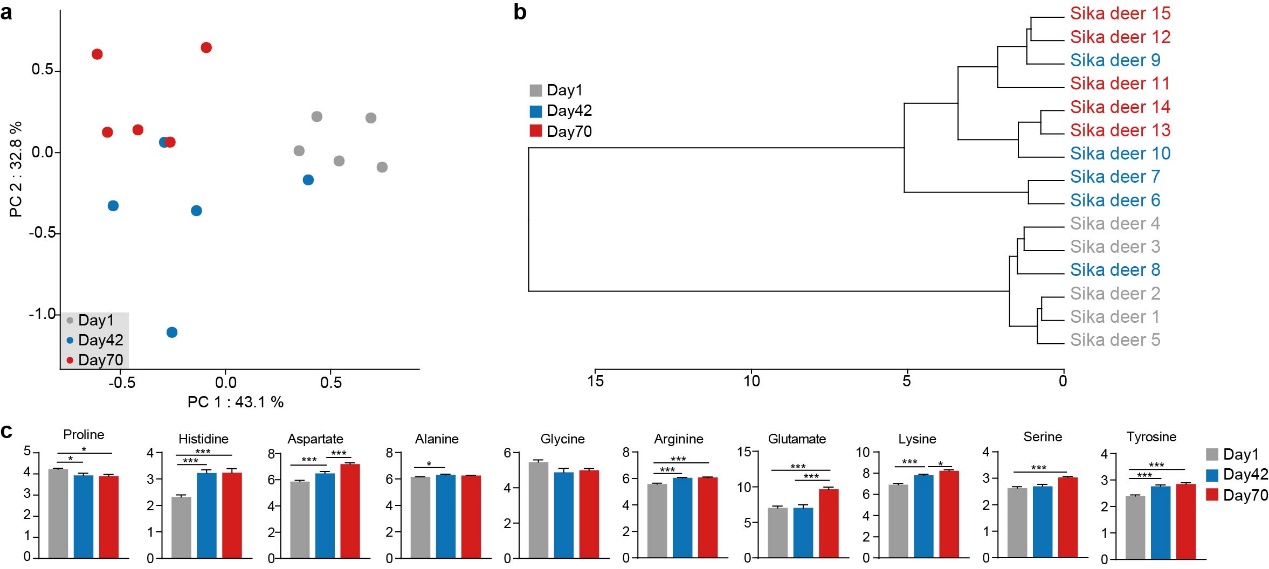


**Figure S2. Amino acid profiles in *longissimus lumborum* (*LL*) of sika deer from birth to postweaning**. **(a)** PCA and hierarchical clustering (**b**) of amino acid profiles in *LL*. Samples are colored by age groups (Day 1: gray, Day 42: blue, and Day 70: red). (**c**) Box plots showing the change of amino acid concentration during early growth period (Concentration=g/100 g). Benjamini-Hochberg-adjusted *P*-values were determined by ANOVA. Bar and whiskers represent the mean ± s.d. *, **, and *** indicate the Benjamini-Hochberg-adjusted *P*-value <0.05, <0.01 and <0.001, respectively.

**
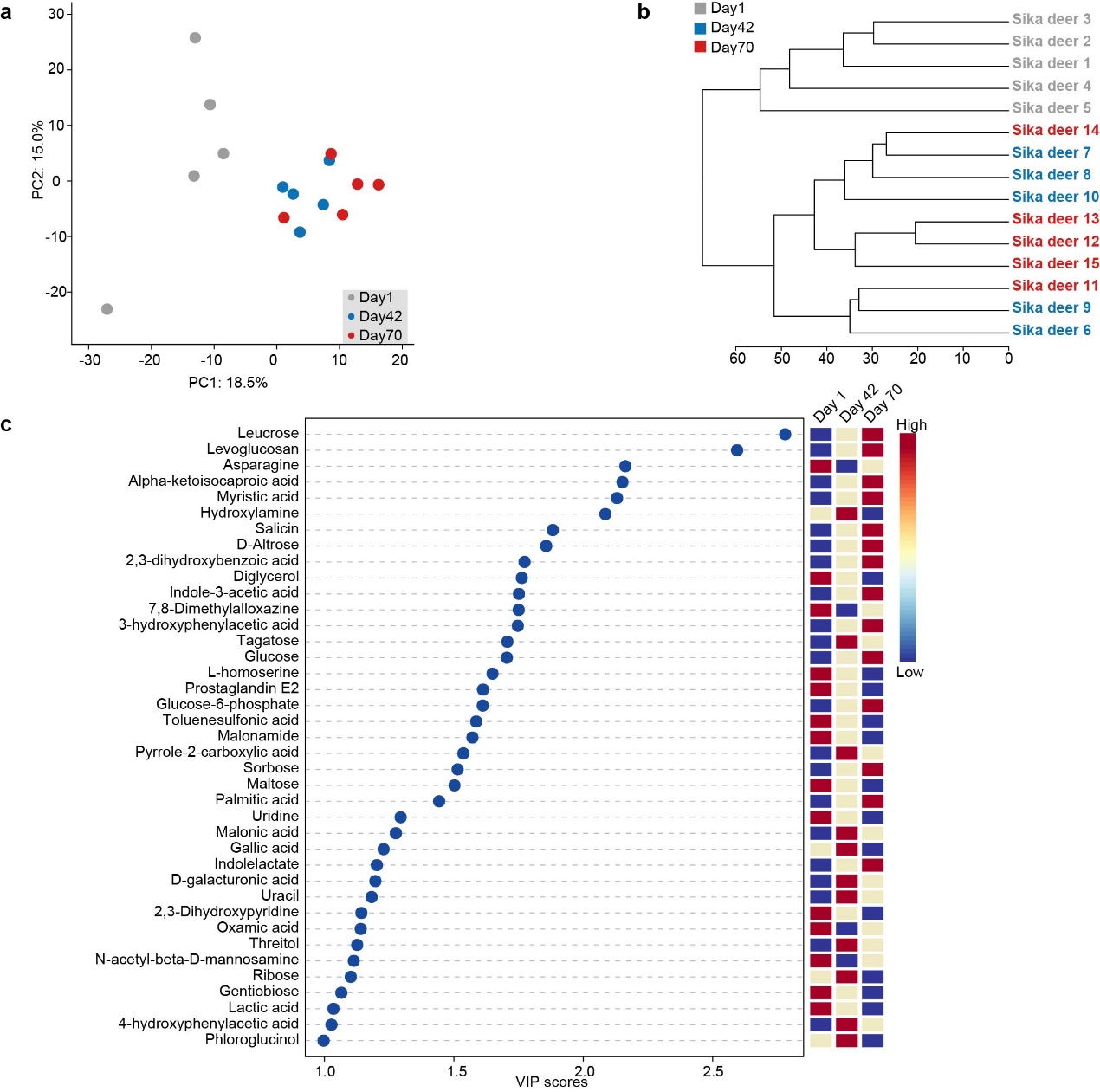
**

**Figure S3. Variational characteristics of serum metabolites in sika deer from birth to postweaning.** (**a**) PCA of all metabolites in serum of 15 sika deer. PCA vector separates samples into age groups, and are colored by gray, blue and red circles, respectively. The metabolites were identified using GC-MS. (**b**) Hierarchical clustering of 15 serum samples based on the identified metabolites. The samples from different age groups were indicated by gray, blue and red text. (**c**) Summary plot showing the most important 39 metabolites in serum ranked based on the VIP scores (>1.0). The heatmap on the right indicate their concentration variations among the three age groups (day 1, day 42 and day 70).


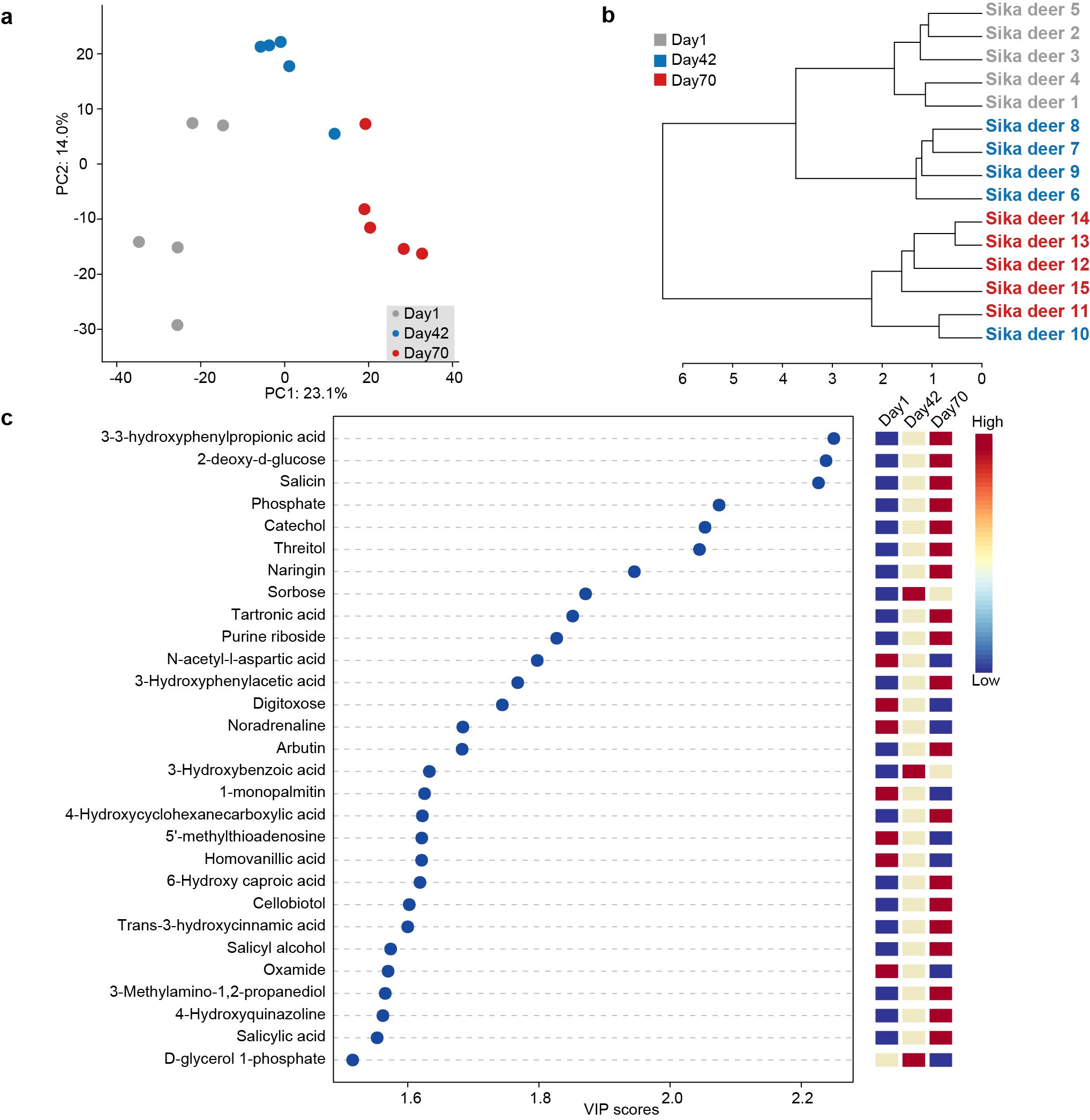


**Figure S4. Urine metabolic profile of sika deer from birth to postweaning.** (**a**) PCA of all urinary metabolites in 15 samples. PCA vector separates samples into age groups. The metabolites were identified using GC-MS. (**b**) Hierarchical clustering of 15 urinary samples based on the identified metabolites. The samples from different age groups were indicated by gray, blue and red text. (**c**) Summary plot showing the most important 29 metabolites in urine ranked based on the VIP scores (>1.0). The heatmap on the right indicate their concentration variations among the three age groups (day 1, day 42 and day 70).

**
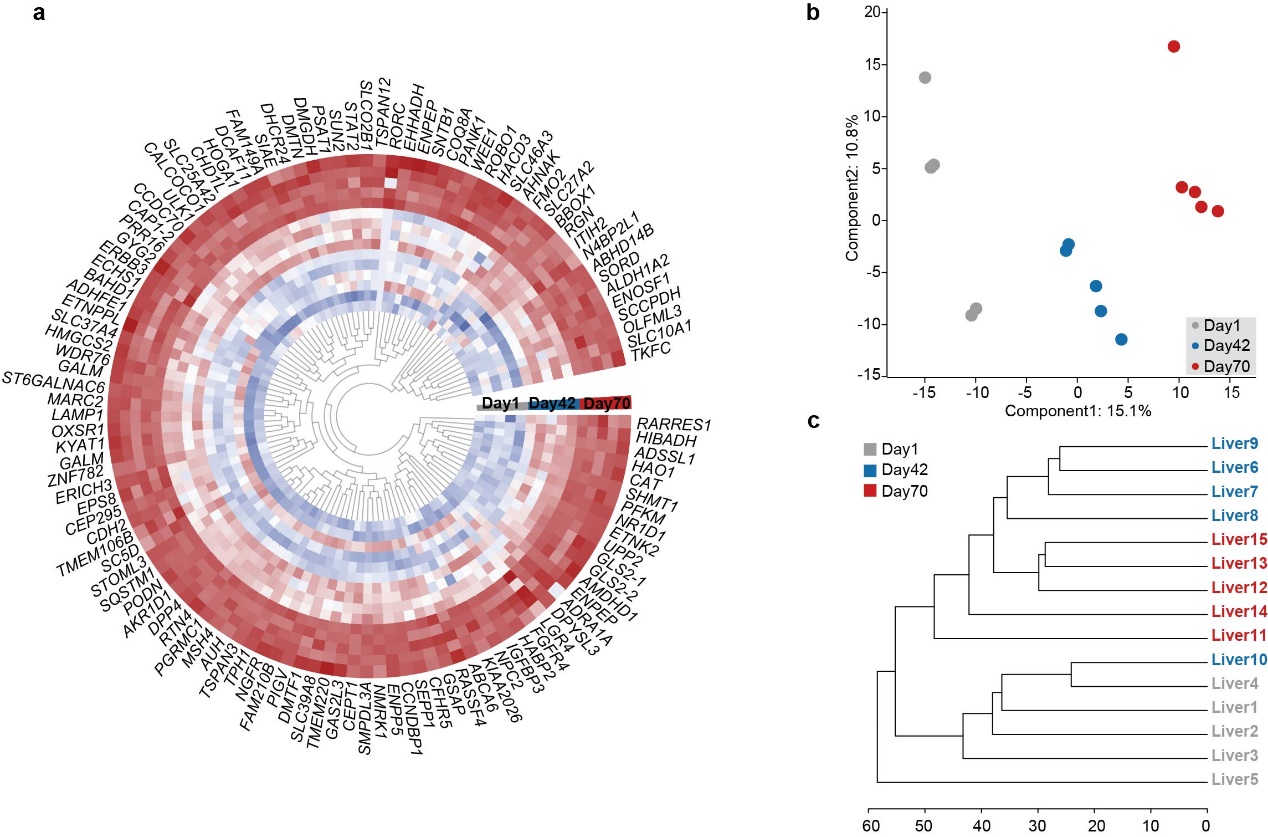
**

**Figure S5. Transcript and metabolic shifts in the liver of sika deer from birth to postweaning.** (**a**) Circular heatmap showing the expression of 144 significantly up-regulated differently expressed genes (DEGs) in liver among the three stages. The samples were colored by gray (day 1), blue (day 42) and red (day 70). Individuals are colored (blue to red) to indicate expression level (low to high). (**b**) PLS-DA showing the variation of hepatic metabolites among the three time points. The samples were colored by gray, blue and red circles. (**c**) Hierarchical clustering of hepatic samples based on the identified metabolites. The samples from different age groups were indicated by gray, blue and red text.


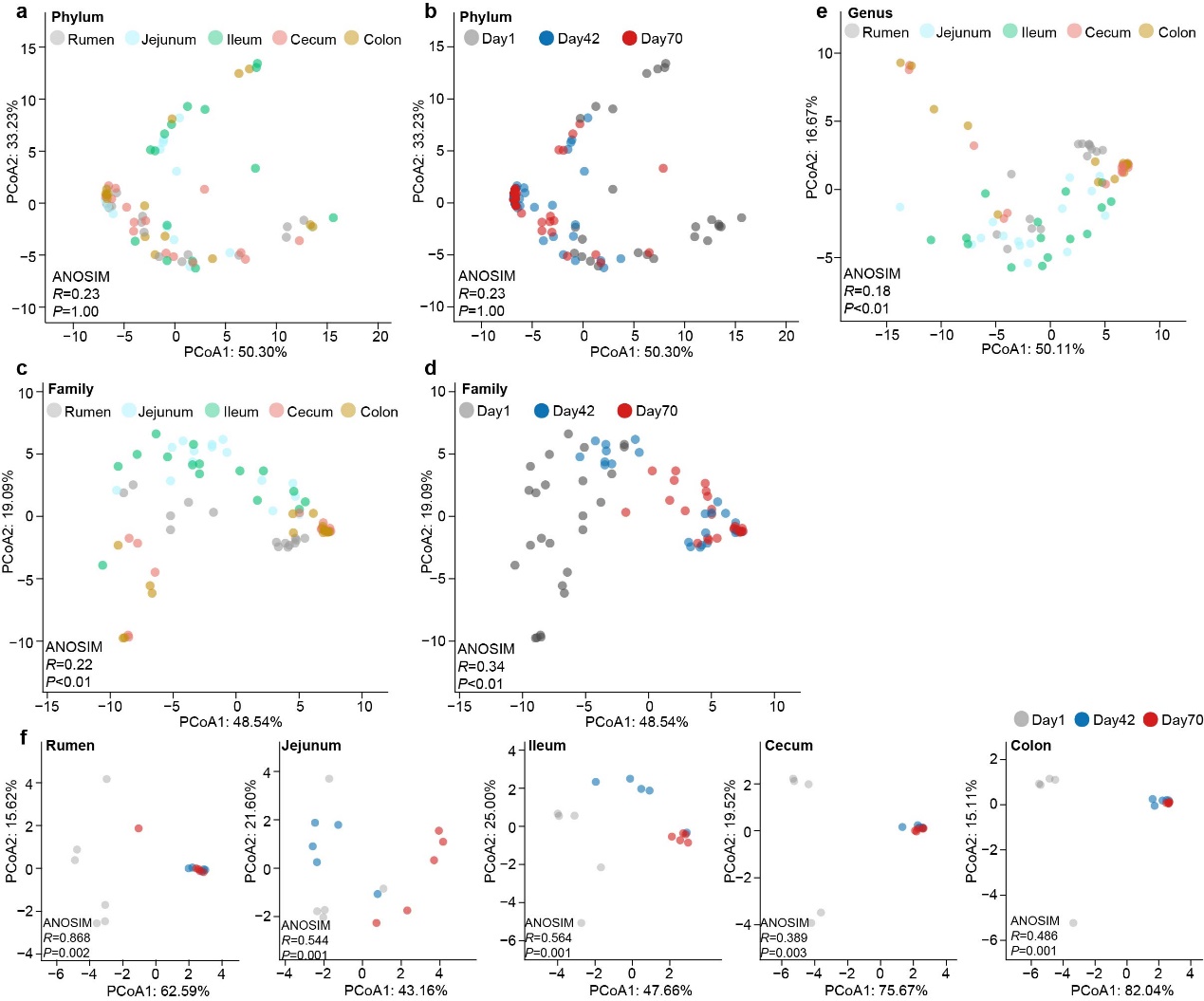


**Figure S6. Variation of GIT microbial community composition from birth to postweaning in sika deer.** PCoA of GIT taxonomic community composition at phylum (**a, b**), family (**c, d**), and genus levels (**e**) based on Bray-Curtis dissimilarity. The samples from different GIT regions were indicated by filling color (rumen: gray, jejunum: light blue, ileum: light green, cecum: pink, and colon: yellow), and from age groups were indicated by gray, blue, and red. (**f**) PCoA of taxonomic community composition in rumen, jejunum, ileum, cecum and colon among the three stages. ANOSIM analysis was used for statistical testing of group similarities. The proportion of variation explained for each axis is given after colon.


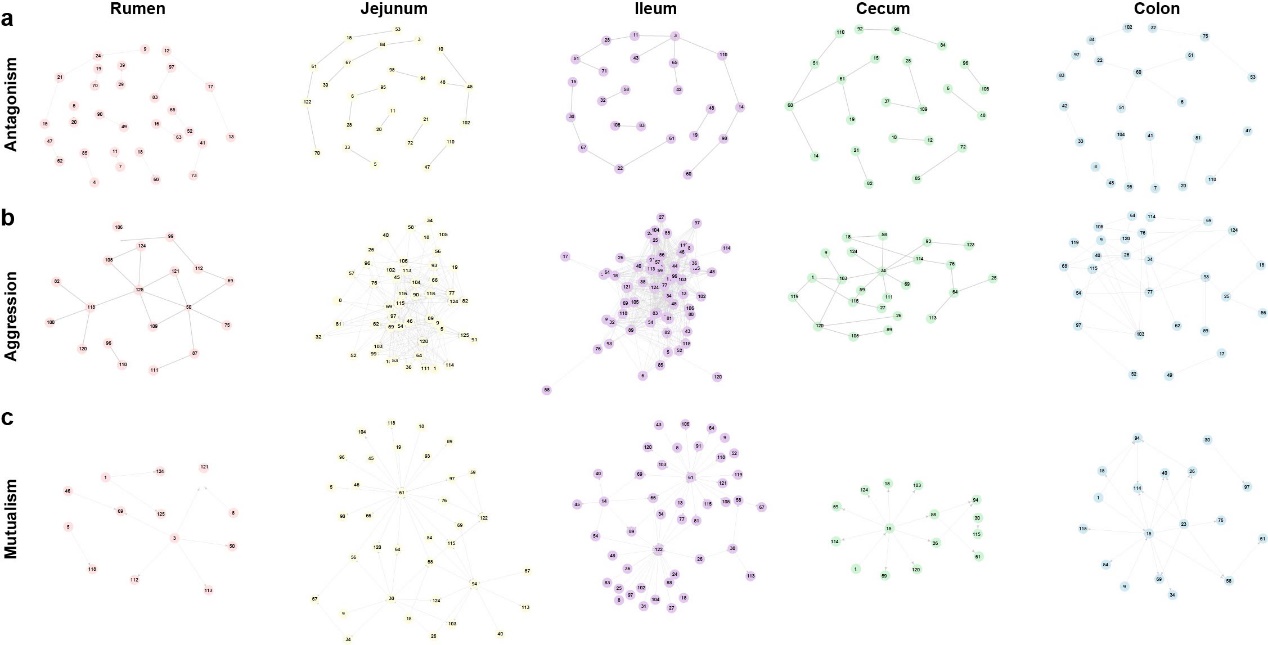


**Figure S7. Microbial interaction networks in the rumen, jejunum, ileum, cecum and colon microbiome of sika deer.** Microbial Z_an_-based antagonism networks (**a**), Z_ag_-based aggression networks (**b**), and Z_mu_-based mutualism networks (**c**) at the genus level within the GIT of sika deer among the three time points.


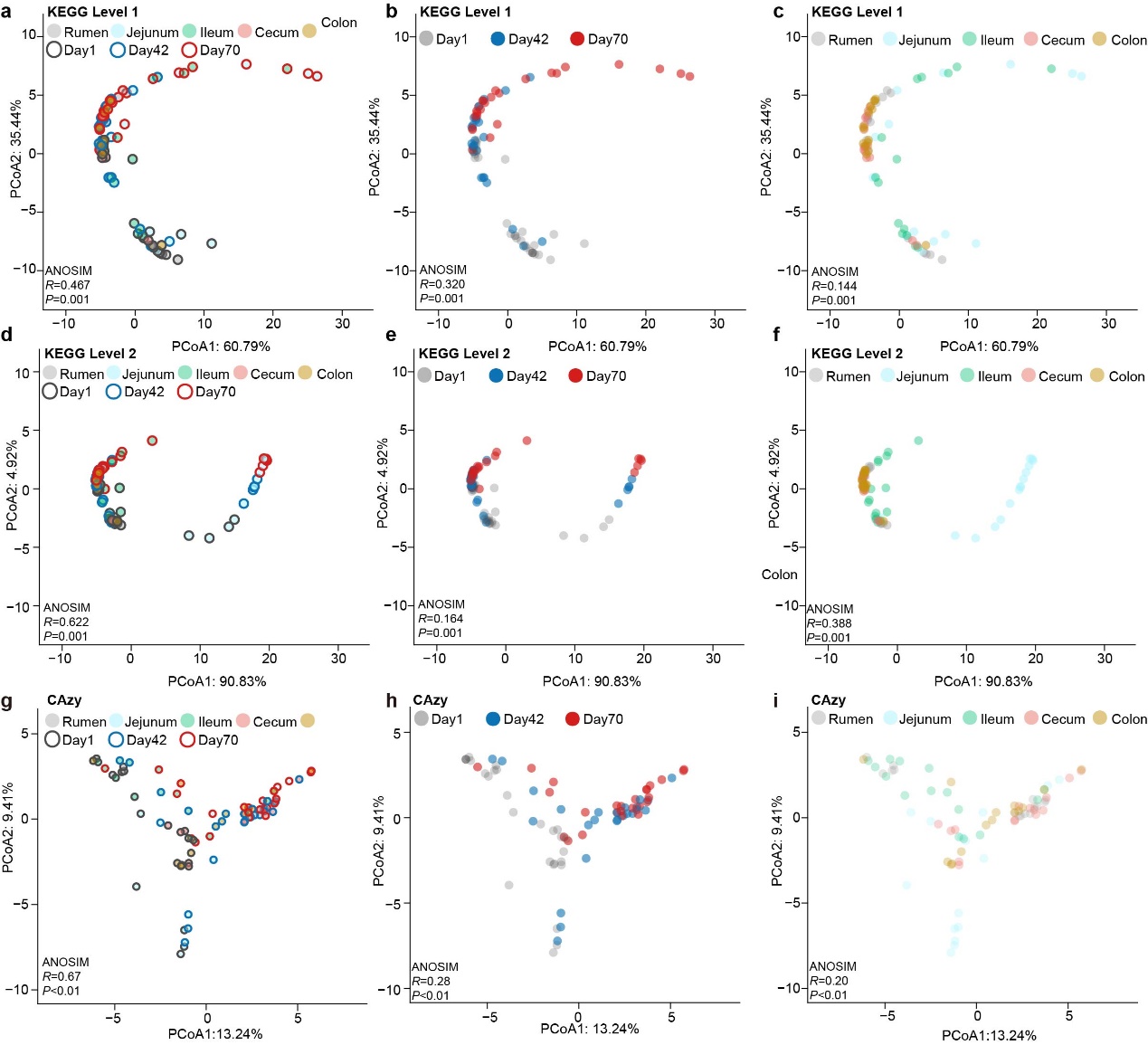


**Figure S8. Changes in the GIT microbial metabolic profiles in sika deer from birth to postweaning.** PCoA of microbial metabolic profiles at KEGG level 1 (**a, b, c**), KEGG level 1 (**d, e, f**), and CAZy levels (**g, h, i**) based on Bray-Curtis dissimilarity. The samples from GIT regions were indicated using filling color (rumen: gray, jejunum: light blue, ileum: light green, cecum: pink, and colon: yellow), and from age groups were indicated by gray, blue, and red. ANOSIM analysis was used for statistical testing of group similarities.


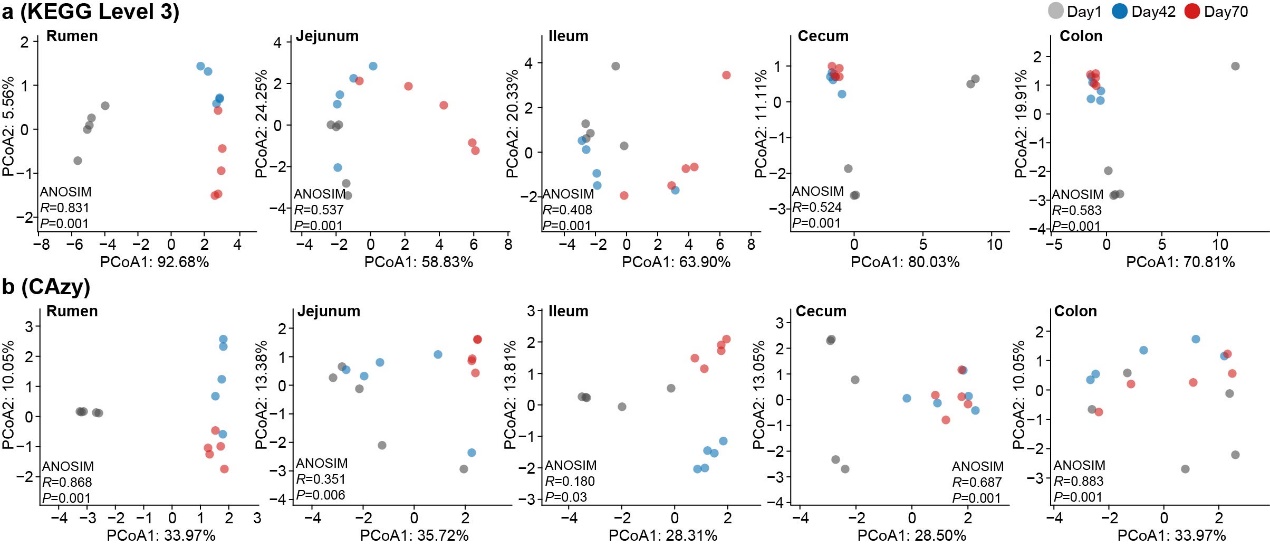


**Figure S9. Changes in KEGG level 3 and CAZy annotations in five GIT regions from birth to postweaning.** PCoA of microbial metabolic profiles at KEGG level 3 (**a**), and CAZy levels (**b**) in rumen, jejunum, ileum, cecum, and colon based on Bray-Curtis dissimilarity. The samples from day 1, day 42 and day 70 were indicated by gray, blue and red circles. ANOSIM analysis was used for statistical testing of group similarities.


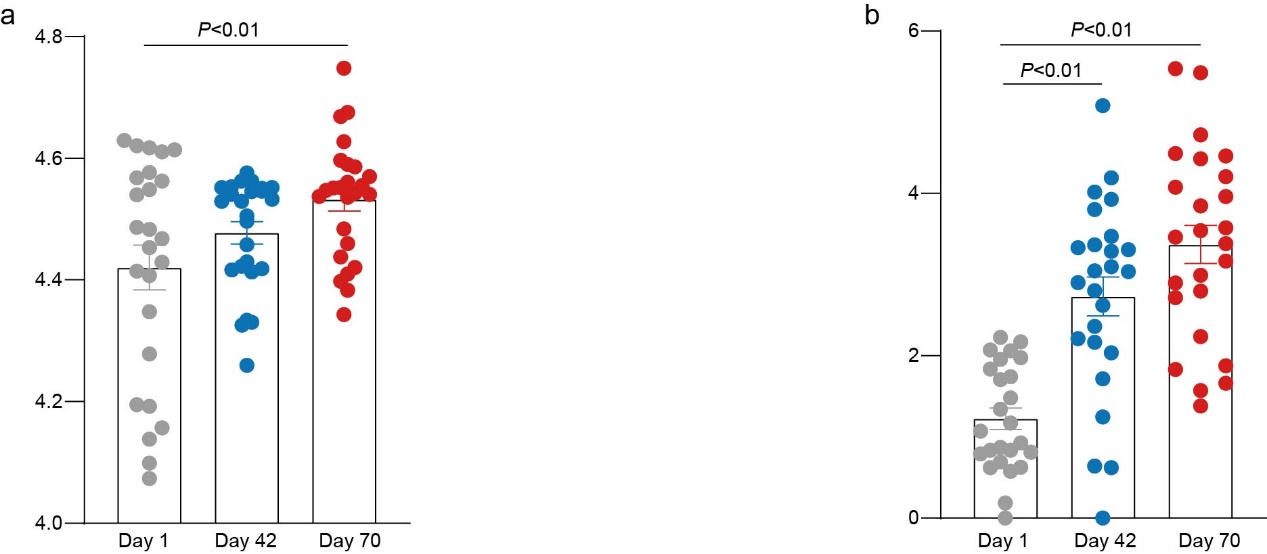


**Figure S10. Comparison of diversity indices for functional annotations generated from the GIT of sika deer over early life development.** Box plot showing the Shannon diversity index of KEGG level 3 (**a**) and CAZy (**b**) from day 1, to days 42 and 70. Benjamini-Hochberg-adjusted *P*-values were determined by ANOVA. Bar and whiskers represent the mean ± s.d..


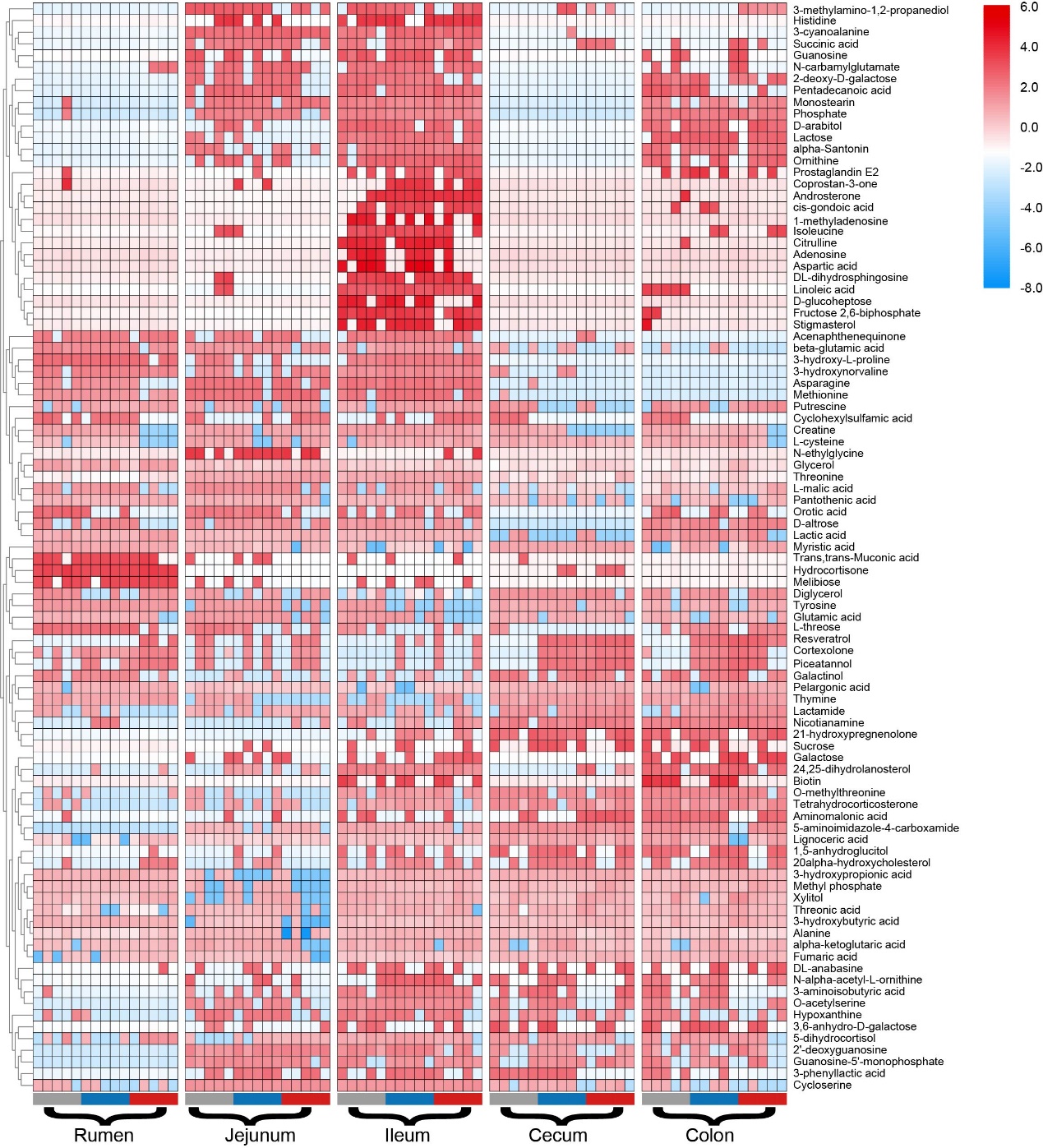


**Figure S11. Heatmap showing the global change of GIT metabolites from birth to postweaning in sika deer.** A total of 93 significant metabolites identified by VIP values (>1), SAM and/or ANOVA methods in each GIT regions. Colors indicate the normalized relative concentration of each metabolite from minimum (blue) to maximum (red). From left to right: rumen, jejunum, ileum, cecum and colon.


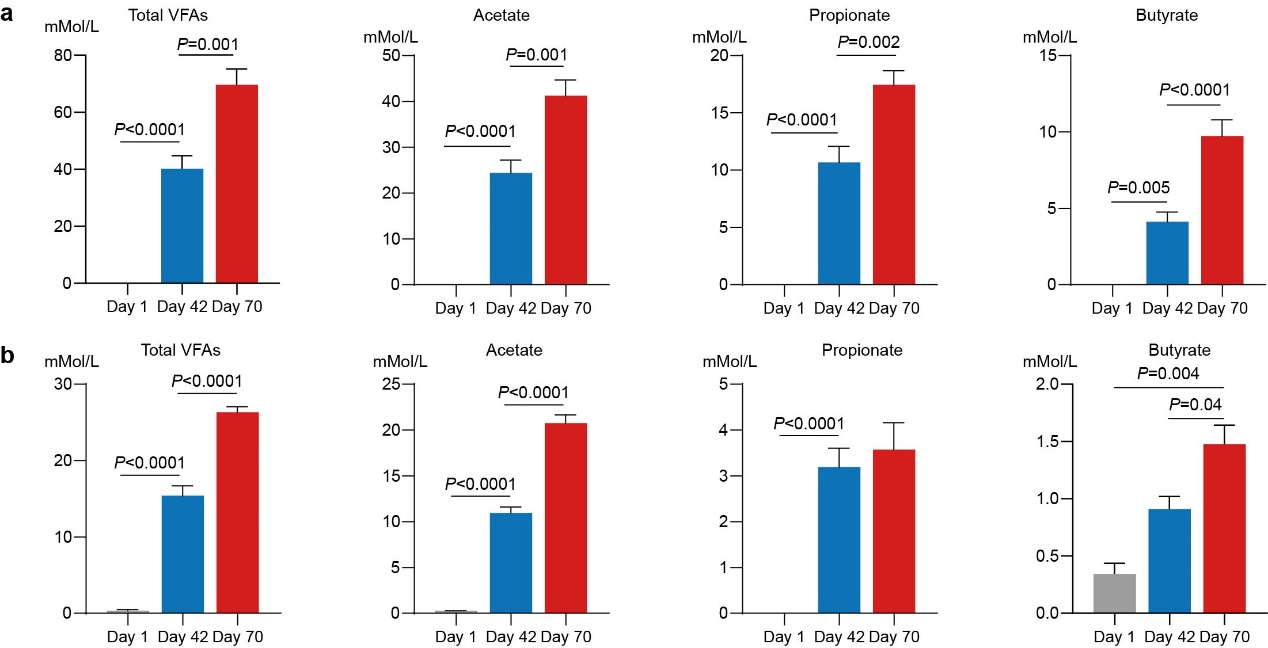


**Figure S12. Comparison of VFA concentrations measured from the rumen (a) and colon (b) of sika deer during early life development stages.**


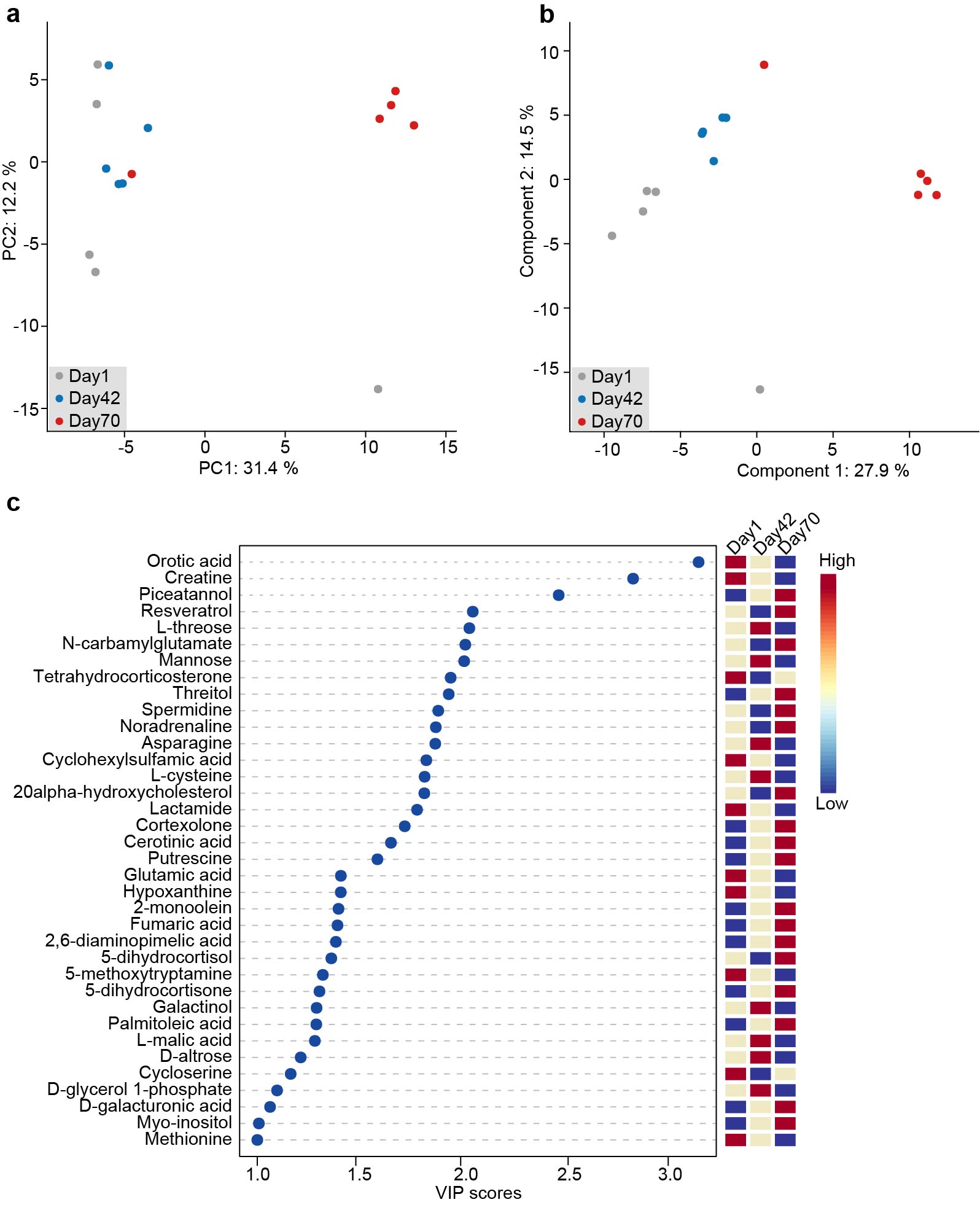


**Figure S13. Metabolic profile in the rumen content of sika deer from birth to postweaning.** (**a**) PCA and (**b**) PLS-DA of all rumen metabolites in 15 samples. The metabolites were identified using GC-MS. The samples from different age groups were indicated by gray, blue and red circles. (**c**) Summary plot showing the most important 36 metabolites in rumen content ranked based on the VIP scores (>1). The heatmap on the right indicate their concentration variations among the three age groups (day 1, day 42 and day 70).


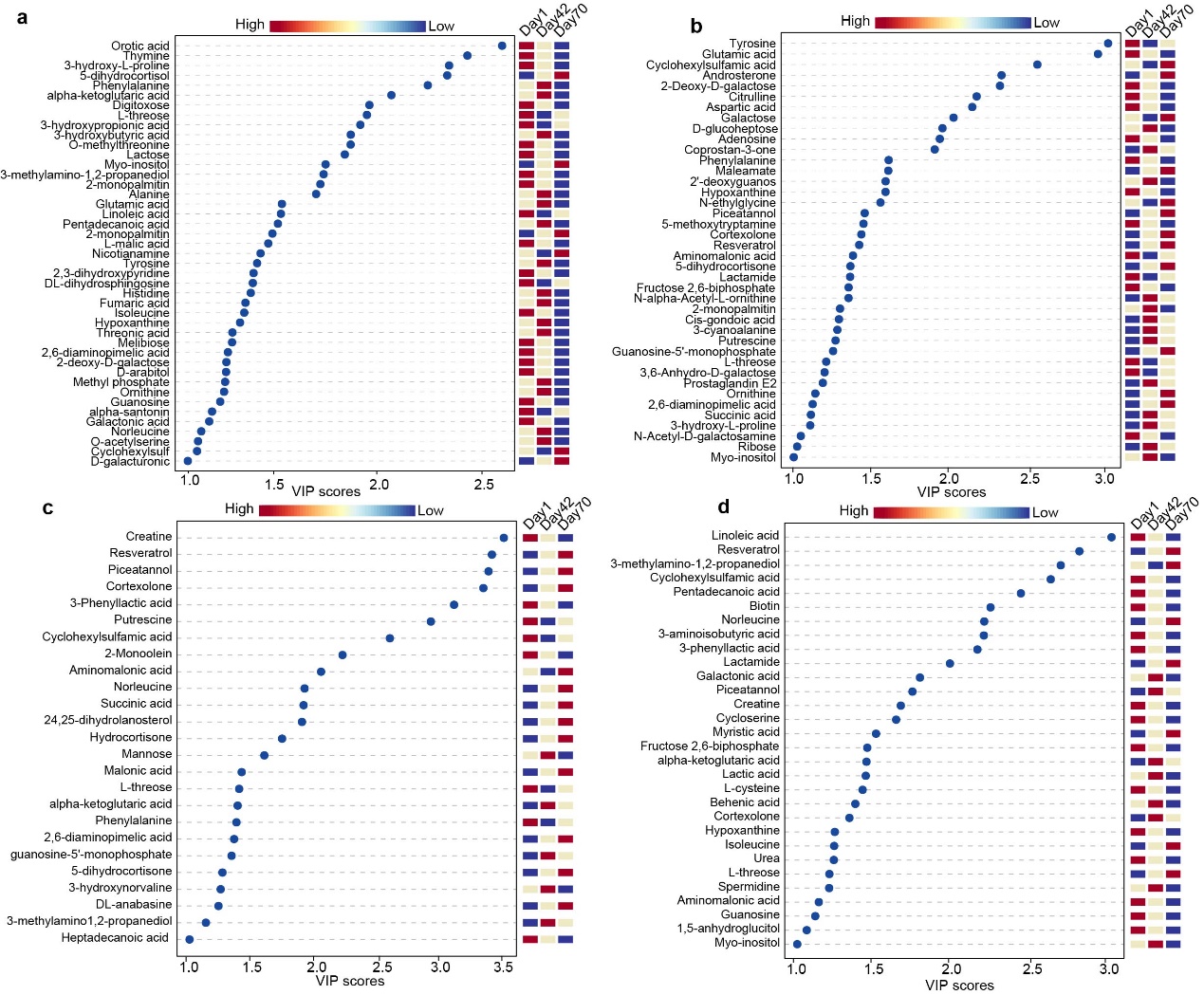


**Figure S14. Significantly changed metabolites detected in the jejunum (a), ileum (b), cecum (c) and colon (d).** The metabolites were ranked based on the VIP scores (>1). The heatmap on the right indicate their concentration variations among the three age groups (day 1, day 42 and day 70).


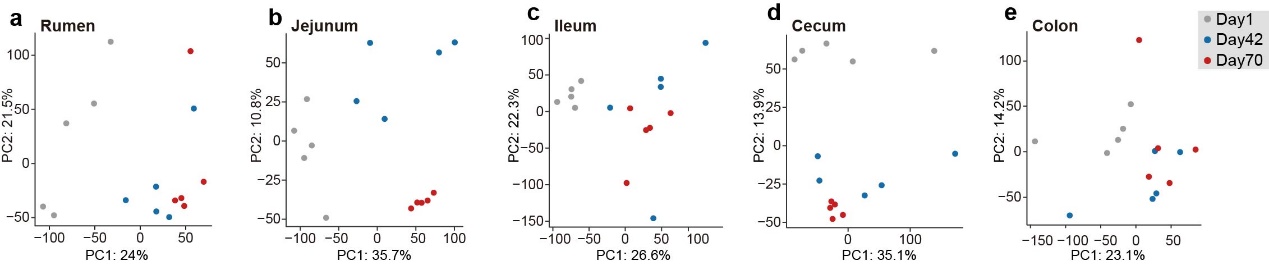


**Figure S15. PCA of all expressed genes in host tissue sampled from the rumen (a), jejunum (b), ileum (c), cecum (d) and colon (e).** The samples from different stages were indicated by gray, blue and red circles.

**
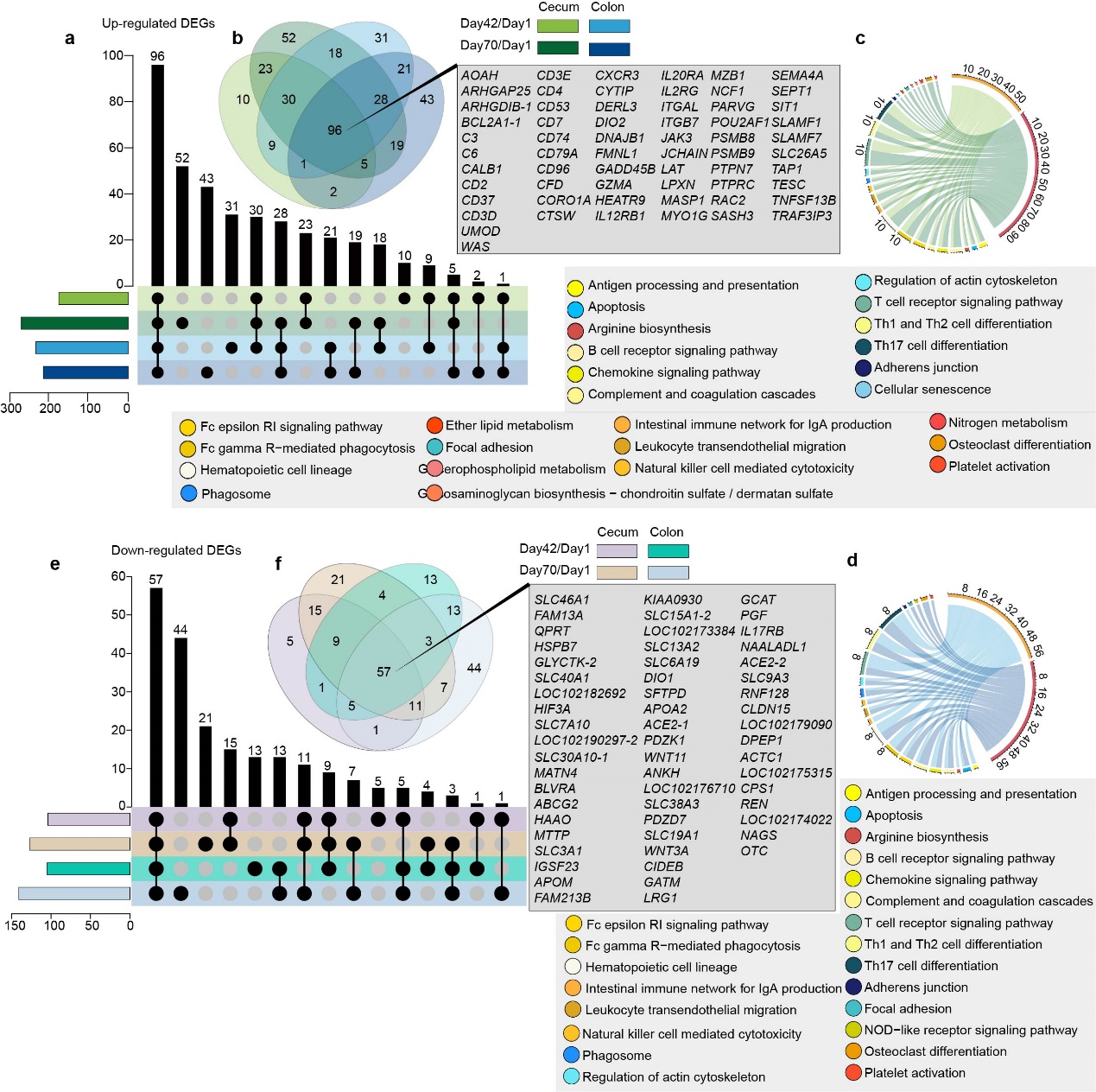
**

**Figure S16. Changes in the cecum and colon epithelium transcriptome from birth to postweaning in sika deer.** Upset and venn diagram showing the significantly and shared up-regulated (**a and b**) and down-regulated DEGs (**e and f**) in cecum and colon epithelium among the comparison of the three stages. A circular plot showing the significantly enriched pathways of the up-regulated DEGs in cecum (**c**) and colon (**d**) epithelium. The DEGs were determined by the fold change ≥ 2 and a Benjamini-Hochberg-adjusted *P*-value < 0.05. The different color curve in circular plot represents the comparisons (Day 42 vs Day 1, and Day 70 vs Day 1). The number at out circle indicate the size of the gene sets in each pathway. The color at out circle indicates the enriched pathways.
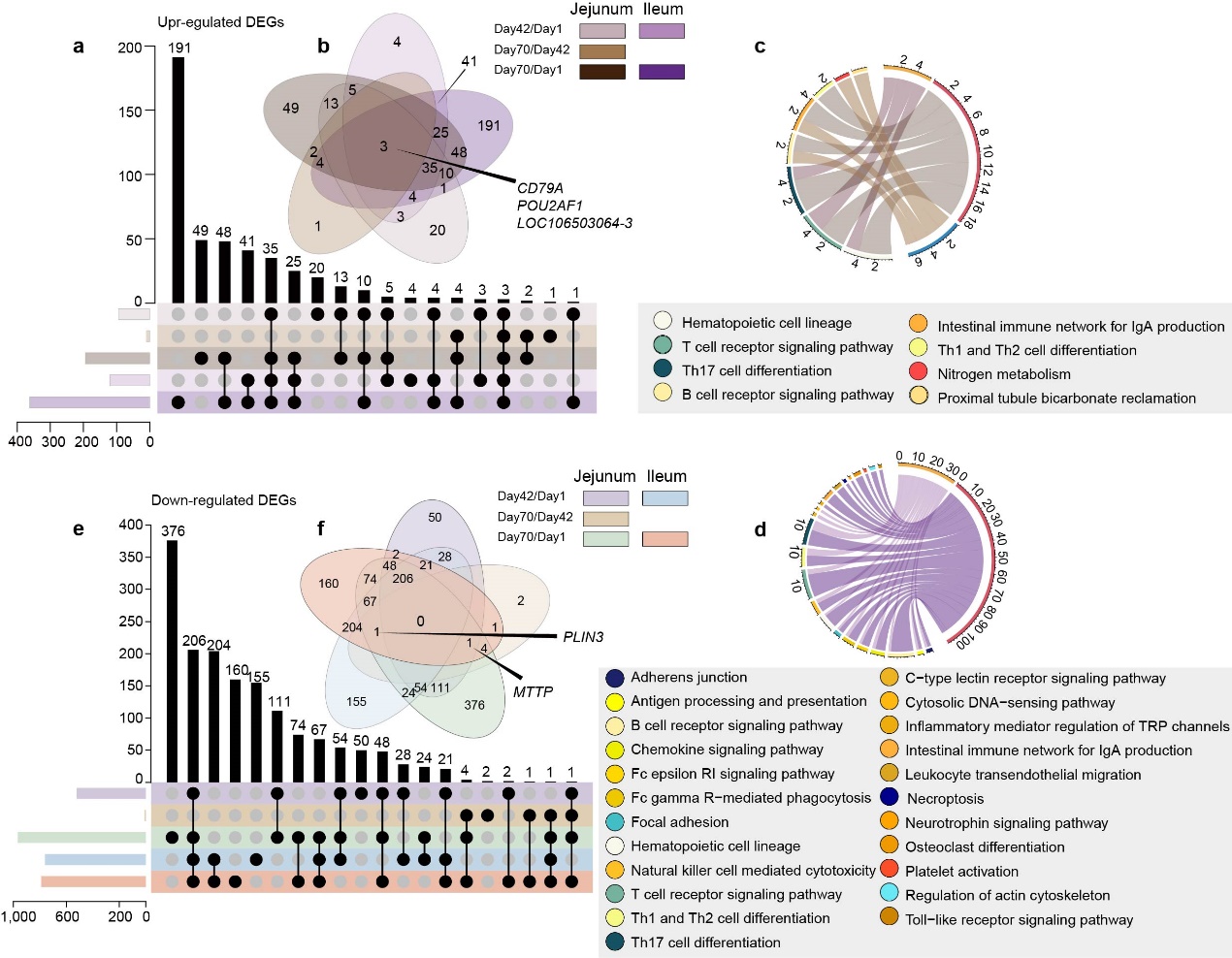


**Figure S17. Changes in the jejunum and ileum epithelium transcriptome from birth to postweaning in sika deer.** Upset and venn diagram showing the significantly and shared up-regulated (**a and b**) and down-regulated DEGs (**e and f**) in jejunum and ileum epithelium among the comparison of the three time points. A circular plot showing the significantly enriched pathways of the up-regulated DEGs in jejunum (**c**) and ileum (**d**) epithelium. The DEGs were determined by the fold change ≥ 2 and a Benjamini-Hochberg-adjusted *P*-value < 0.05. The different color curve in circular plot represents the comparisons (Day 42 vs Day 1, Day 70 vs Day 42, and Day 70 vs Day 1). The number at out circle indicate the size of the gene sets in each pathway. The color at out circle indicates the enriched pathways.

**
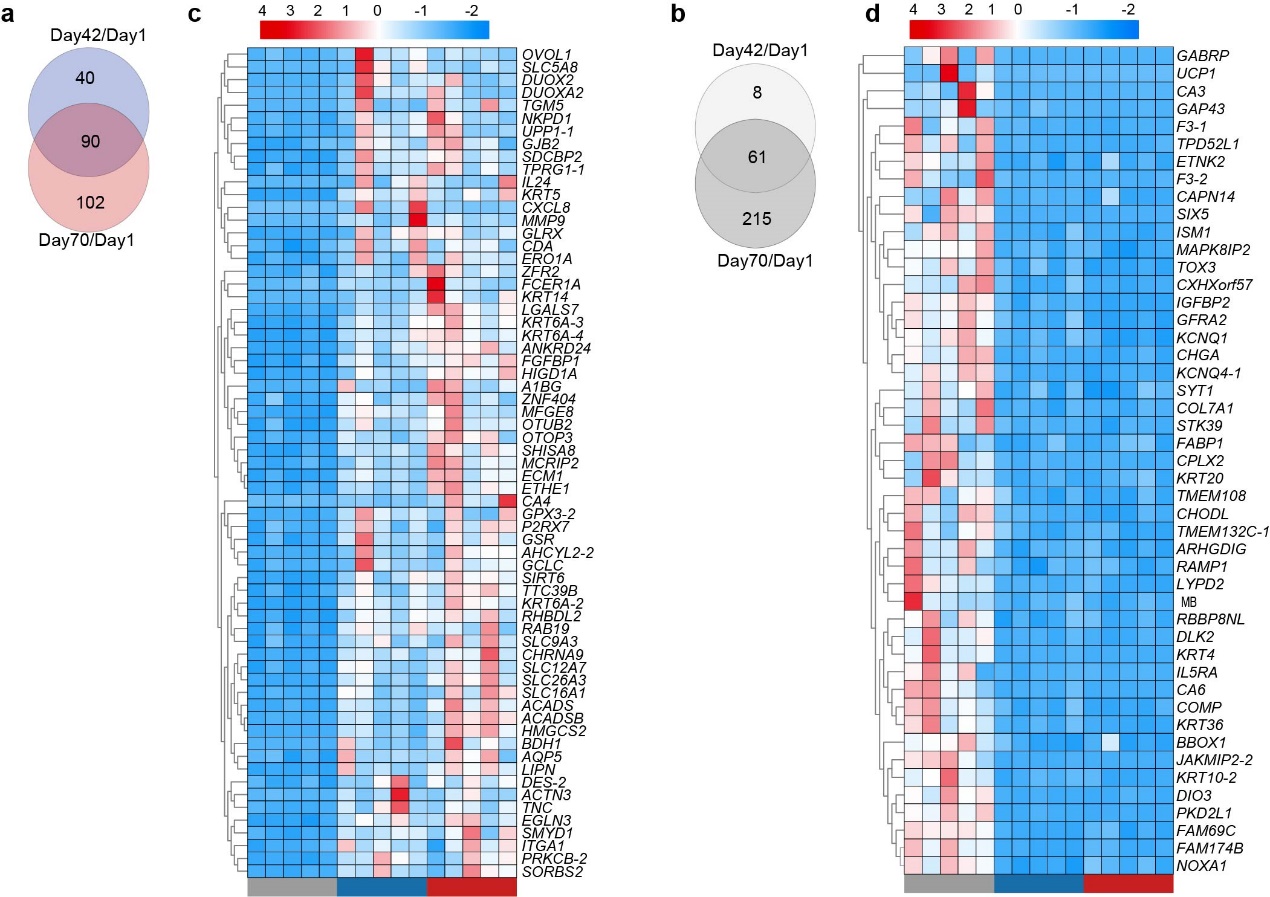
**

**Figure S18. Transcriptomic differences observed in the rumen epithelium in sika deer from birth to postweaning.** Venn diagram showing the number of significantly up- (**a**) and down-regulated (**b**) DEGs in rumen epithelium. Heat map revealing the significantly up- (**c**) and down-regulated (**d**) DEGs in the rumen epithelium among the three time points. The samples were colored by gray (day 1), blue (day 42) and red (day 70). Individuals are colored (blue to red) to indicate expression level (low to high).
