# Supplementary material for: Integrated multi-omics of the gastrointestinal microbiome and ruminant host reveals metabolic adaptation underlying early life development
Source: Microbiome. 2022 Dec 12;10:222. doi: 10.1186/s40168-022-01396-8 (PMC9743514; doi:10.1186/s40168-022-01396-8)
Supplement: Supplementary file 6 — Additional file 5. Supplementary Discussion. [file 40168_2022_1396_MOESM5_ESM.docx]

**Supplementary Discussion**

**Significant change of fatty acid profiles in body composition, serum and urine**

A decreased trend of N-acetyl-L-aspartic acid (N-acetylaspartate) was observed in urine (**Fig. 1h**), which is considered a critical energy metabolite for lipid turnover and whole-body energy homeostasis [1]. In addition, the concentrations of caffeic acid and its derivative (trans-3-hydroxycinnamic acid) in urine (**Fig. 1h**), and leucrose in serum (**Fig. 1g**) increased with age, both of which have been linked to increases in fatty acid β-oxidation, and significantly inhibition of fatty acid accumulation [2, 3]. Taken together, we interpret this signature as an indicator that the increased efficiency of fatty acid oxidation resulted in the change of body composition phenotype that was featured during early life for these animals.

**Global transcriptome and metabolome revealing change of hepatic fatty acid metabolism**

We found the specifically up-regulated 839 differently expressed genes (DEGs, Day 70 and Day 42) were enriched in lysosome, endocytosis, mitophagy, and cholesterol metabolism in hepatic function from preweaning to postweaning, which involved in the genes related to cholesterol metabolism including *Lipa*, *Lamp2*, *Ldlr*, *Npc2*, *Acaa2*, *Acsl*, and *Cpt1* (**Fig. 2c and Table S2**). Lysosomes promote lipid catabolism and transport; both of which are critical in maintaining cellular lipid homeostasis [4]. The low-density lipoprotein receptor (*Ldlr*) is involved in the control of blood cholesterol by clearing cholesterol-laden low-density lipoprotein (LDL) particles via endocytosis [5]. LDL particles are attacked by the lysosomal acid lipase type A (*Lipa*), a hydrolase that de-esterifies the cholesterol and triglyceride molecules releasing free fatty acids, which are then transported to mitochondria for FA β-oxidation to produce ATP [6]. LAMP2 is one of the limiting lysosomal membrane glycoproteins, which is more critical for the cholesterol transport, and affect NPC2, which has been proposed to act in cholesterol efflux by binding cholesterol in the lysosomal lumen and delivering it to other proteins [7]. Carnitine palmitoyltransferase 1 (*Cpt1*) is one key carnitine-dependent cycle enzyme that plays a role in the transportation of long-fatty acids into the mitochondria for FA β-oxidation [8]. Acetyl-CoA acyltransferase 2 (*Acaa2*) encoding protein catalyzes the last step of the mitochondrial FA β-oxidation spiral [9]. Moreover, bile acids synthesized in the liver from cholesterol and are subsequently secreted across the hepatic canalicular membrane by the ATP binding cassette transporter (*Abcb11*) [10]. These results provide evidence that cholesterol transport and metabolism for energy production via FA β-oxidation in liver is likely enhanced from preweaning to postweaning for juvenile ruminant, which could represent a significant source for the changed fatty acids composition of body.

**Immediately diverse and regional microbe colonization across GIT after birth**

In this study, the detection of protozoal species *Eimeria* spp. in the small intestine (jejunum and ileum) did not agree with previous finding in calves dominated by *Cryptosporidium* spp. [11]. The genus *Eimeria* has been shown to be specific for each host where they develop in the small and large intestine and could particularly cause infection in young animals [12].

We found that the specific core microbiota for each distinct GIT region such as the rumen (*Lactobacillus* spp.), small intestine (*Lactobacillus* spp.), and large intestine (*Bacteroides* spp.), showed the most noticeable and significant decrease at days 42 and 70 when compared to day 1 (**Fig. 3f**). Several species of the genus *Lactobacillus* are known to ferment sugars to produce lactate, and their colonization in the ileum has been shown to play a role in regulating immune responses [11]. Select *Bacteroidetes* spp. encode the genes that are used to harvest host mucus glycans during human milk oligosaccharides consumption [13]. These results suggested a determined role of each GIT regions in selecting the pioneer microbial community. Interestingly, we found the higher prevalence of *T7virus* (phage) and *Yesrinia* spp. in ileum at day 1. Recent studies revealed that the *T7virus* was the common phages infecting *Yersinia* species [14]. These findings indicated that the role of bacteria-phage interaction in affecting microbial succession in ileum needs further examination.

**The altruism type driven GIT microbiota assembles from birth to postweaning**

To further understand the mechanisms behind the microbial community shifts observed in our data, we integrated behavioral eco-evolutionary theory to reflect the possible types of microbe-microbe interactions, including mutualism, antagonism, aggression, and altruism [15]. The results showed that an altruism network was the dominant interaction type in rumen, jejunum, ileum, cecum, and colon (**Fig. 4 and S7**), suggesting that a cooperative manner is important for GIT microbial community assembly at early life. Of the hub microbes, *K1gvirus* (*Kagunavirus*, Siphoviridae family) was presented in networks of each GIT region and *Acanthamoeba* spp. (protozoa) in rumen and small intestine (**Fig. 4**). Results of human infants has also revealed that the gut microbiome shifted from a high bacteriophage-low bacterial diversity community at birth toward a low bacteriophage-high bacterial diversity community by 24 months of age [16]. The viral colonization in early life is stepwise, with the first phase characterized by the induction of temperate prophages from pioneer bacteria, and a second phase involving colonization with viruses that replicate in human cells, which is modulated by breastfeeding [17]. The processive degradation of external polysaccharides by the genus *Kagunavirus* via lytic infection of bacteria play important roles to evolve new host specificities [18]. *Acanthamoeba* may act as a ‘Trojan horse’ facilitating bacterial transmission in human gut [19]. Although we could not illuminate the source of phages and protozoa, these data suggested the potential interaction between phage and bacteria, and the interaction of protozoa and bacteria is hypothesized to help the microbial colonization in GIT of juvenile ruminant.

**Transcriptomic analysis of GIT epithelium of sika deer after birth**

To further explore the adaption and functional changes of the GIT epithelium during growth, we conducted RNA-seq based transcriptomics. We observed that *Pou2af1* was specially up-regulated in both the jejunum and ileum (**Fig. S17b**), which is necessary for B-cell response to antigens and required for germinal centers formation [20]. The intestinal immune network for IgA production was also significantly enriched in the ileum (**Fig. 7f and S17d**) in comparison with the jejunum (**Fig. S17c**) and cecum (**Fig. S18c**), which involved the genes *Itgb7*, *Itga4*, *Tnfrsf13c*, *Ccr9*, *Tnfrsf13B*, and *Tnfsf13b*. *Tnfsf13* involved in B cell and immunoglobulin A immune system function, whose knockout showed the impaired IgA antibody responses to mucosal immunization [21]. ITGB7 protein heterodimerizes with ITGA4 (integrin alpha 4) protein to form the gut-homing heterodimer LPAM1 (lymphocyte PP adhesion molecule, α4β7), which reduce the gut-homing efficiency of mature IgA plasma cells when the expression decreased [22]. Our findings further address the importance of regulating IgA production in the ileum after weaning. However, this conclusion is inconsistent with previous finding that the jejunum is the site for greater secretion of IgA than the ileum of preweaning calf [23], which is likely related to different physiology stage (pre- and post-weaning).

We also found that nitrogen metabolism (*Ca3*, *Ca7*, *Ca12*) were also enriched in the ileum and large intestine (**Fig. 7f and S17d, S18c-d**). The gene *Ca*s encoding carbonic anhydrases, that catalyze the hydration of carbon dioxide and dehydration of carbonic acid, which were efficiently activated by tyrosine [24]. Of note, the features of the methane metabolism pathway inferred in producing carbon dioxide were significantly enriched, while the concentration of tyrosine increased postweaning in the colon (**Fig. 6d**). It is also observed that linoleic acid accumulated during necroptosis [25], which were also specially increased in the ileum (**Fig. S11 and S14d**). Thus, we concluded that the metabolism of fatty acid and amino acid may play role in necroptosis and apoptosis, which could affect epithelium development and function of ileum and colon, respectively.

**References**

1. Hofer DC, Zirkovits G, Pelzmann HJ, Huber K, Pessentheiner AR, Xia W, Uno K, Miyazaki T, Kon K, Tsuneki H *et al*: **N-acetylaspartate availability is essential for juvenile survival on fat-free diet and determines metabolic health**. *FASEB J* 2019, **33**(12):13808-13824.

2. Alam MA, Subhan N, Hossain H, Hossain M, Reza HM, Rahman MM, Ullah MO: **Hydroxycinnamic acid derivatives: a potential class of natural compounds for the management of lipid metabolism and obesity**. *Nutr Metab* 2016, **13**(1):27.

3. Lee J, Kim E, Kim Y, Yoo S-H: **Leucrose, a sucrose isomer, suppresses hepatic fat accumulation by regulating hepatic lipogenesis and fat oxidation in high-fat diet-induced obese mice**. *J Cancer Prev* 2018, **23**(2):99-106.

4. Thelen AM, Zoncu R: **Emerging roles for the lysosome in lipid metabolism**. *Trends Cell Biol* 2017, **27**(11):833-850.

5. Jeon H, Blacklow SC: **Structure and physiologic function of the low-density lipoprotein receptor**. *Annu Rev Biochem* 2005, **74**(1):535-562.

6. Singh R, Cuervo Ana M: **Autophagy in the cellular energetic balance**. *Cell Metab* 2011, **13**(5):495-504.

7. Schneede A, Schmidt CK, Hölttä-Vuori M, Heeren J, Willenborg M, Blanz J, Domanskyy M, Breiden B, Brodesser S, Landgrebe J *et al*: **Role for LAMP-2 in endosomal cholesterol transport**. *J Cell Mol Med* 2011, **15**(2):280-295.

8. Ogawa E, Kanazawa M, Yamamoto S, Ohtsuka S, Ogawa A, Ohtake A, Takayanagi M, Kohno Y: **Expression analysis of two mutations in carnitine palmitoyltransferase IA deficiency**. *J Hum Genet* 2002, **47**(7):342-347.

9. Cao W, Liu N, Tang S, Bao L, Shen L, Yuan H, Zhao X, Lu H: **Acetyl-Coenzyme A acyltransferase 2 attenuates the apoptotic effects of *BNIP3* in two human cell lines**. *Biochim Biophys Acta Gen Subj* 2008, **1780**(6):873-880.

10. Henkel AS, Gooijert KER, Havinga R, Boverhof R, Green RM, Verkade HJ: **Hepatic overexpression of *Abcb11* in mice promotes the conservation of bile acids within the enterohepatic circulation**. *Am J Physiol Gastrointest Liver Physiol* 2012, **304**(2):G221-G226.

11. Malmuthuge N, Liang G, Griebel PJ, Guan LL: **Taxonomic and functional compositions of the small intestinal microbiome in neonatal calves provide a framework for understanding early life gut health**. *Appl Environ Microbiol* 2019, **85**(6):e02534-02518.

12. Chartier C, Paraud C: **Coccidiosis due to *Eimeria* in sheep and goats, a review**. *Small Ruminant Res* 2012, **103**(1):84-92.

13. Marcobal A, Barboza M, Sonnenburg Erica D, Pudlo N, Martens Eric C, Desai P, Lebrilla Carlito B, Weimer Bart C, Mills David A, German JB *et al*: ***Bacteroides* in the infant gut consume milk oligosaccharides via mucus-utilization pathways**. *Cell Host Microbe* 2011, **10**(5):507-514.

14. Salem M, Skurnik M: **Genomic characterization of sixteen *Yersinia* enterocolitica-infecting *Podoviruses* of pig origin**. *Viruses* 2018, **10**(4):174.

15. Jiang L, Liu X, He X, Jin Y, Cao Y, Zhan X, Griffin CH, Gragnoli C, Wu R: **A behavioral model for mapping the genetic architecture of gut-microbiota networks**. *Gut Microbes* 2020:1-15.

16. Lim ES, Zhou Y, Zhao G, Bauer IK, Droit L, Ndao IM, Warner BB, Tarr PI, Wang D, Holtz LR: **Early life dynamics of the human gut virome and bacterial microbiome in infants**. *Nat Med* 2015, **21**(10):1228-1234.

17. Liang G, Zhao C, Zhang H, Mattei L, Sherrill-Mix S, Bittinger K, Kessler LR, Wu GD, Baldassano RN, DeRusso P *et al*: **The stepwise assembly of the neonatal virome is modulated by breastfeeding**. *Nature* 2020, **581**(7809):470-474.

18. Leiman PG, Battisti AJ, Bowman VD, Stummeyer K, Mühlenhoff M, Gerardy-Schahn R, Scholl D, Molineux IJ: **The structures of *Bacteriophages* K1E and K1-5 explain processive degradation of polysaccharide capsules and evolution of new host specificities**. *J Mol Biol* 2007, **371**(3):836-849.

19. Khan NA: ***Acanthamoeba* : biology and increasing importance in human health**. *FEMS Microbiol Rev* 2006, **30**(4):564-595.

20. Corcoran L, Emslie D, Kratina T, Shi W, Hirsch S, Taubenheim N, Chevrier S: ***Oct2* and *Obf1* as facilitators of B:T cell collaboration during a humoral immune response**. *Front Immunol* 2014, **5**(108).

21. Castigli E, Scott S, Dedeoglu F, Bryce P, Jabara H, Bhan AK, Mizoguchi E, Geha RS: **Impaired IgA class switching in APRIL-deficient mice**. *Proc Natl Acad Sci U.S.A.* 2004, **101**(11):3903-3908.

22. Pilarowski GO, Cazares T, Zhang L, Benjamin JS, Liu K, Jagannathan S, Mousa N, Kasten J, Barski A, Lindsley AW *et al*: **Abnormal peyer patch development and B-cell gut homing drive IgA deficiency in Kabuki syndrome**. *J Allergy Clin Immunol* 2020, **145**(3):982-992.

23. Liang G, Malmuthuge N, Bao H, Stothard P, Griebel PJ, Guan LL: **Transcriptome analysis reveals regional and temporal differences in mucosal immune system development in the small intestine of neonatal calves**. *BMC Genomics* 2016, **17**(1):602-602.

24. Vullo D, Nishimori I, Scozzafava A, Supuran CT: **Carbonic anhydrase activators: activation of the human cytosolic isozyme III and membrane-associated isoform IV with amino acids and amines**. *Bioorg Med Chem Lett* 2008, **18**(15):4303-4307.

25. Parisi LR, Li N, Atilla-Gokcumen GE: **Very long chain fatty acids are functionally involved in necroptosis**. *Cell Chem Biol* 2017, **24**(12):1445-1454.e1448.
